# Pillar data-acquisition strategies for cryo-electron tomography of beam-sensitive biological samples

**DOI:** 10.1107/S2059798324004546

**Published:** 2024-06-03

**Authors:** James M. Parkhurst, Trond Varslot, Maud Dumoux, C. Alistair Siebert, Michele Darrow, Mark Basham, Angus Kirkland, Michael Grange, Gwyndaf Evans, James H. Naismith

**Affiliations:** a Rosalind Franklin Institute, Harwell Science and Innovation Campus, Didcot OX11 0FA, United Kingdom; b Diamond Light Source, Harwell Science and Innovation Campus, Didcot OX11 0DE, United Kingdom; c Thermo Fisher Scientific, Vlastimila Pecha, Brno, Czechia; d Electron Physical Science Imaging Centre, Diamond Light Source, Harwell Science and Innovation Campus, Didcot OX11 0DE, United Kingdom; e Department of Materials, University of Oxford, Parks Road, Oxford OX1 3PH, United Kingdom; f Division of Structural Biology, University of Oxford, Roosevelt Drive, Oxford OX3 7BN, United Kingdom; Institute of Integrative Biology, University of Liverpool, United Kingdom

**Keywords:** cryo-electron tomography, cryo-ET, multislice image simulation, data-collection strategy, reconstruction, alignment, beam-sensitive specimens

## Abstract

For cryo-electron tomography (cryo-ET) of beam-sensitive biological specimens, a planar sample geometry is typically used. As the sample is tilted, the effective thickness of the sample along the direction of the electron beam increases and the signal-to-noise ratio concomitantly decreases, limiting the transfer of information at high tilt angles. In addition, the tilt range where data can be collected is limited by a combination of various sample-environment constraints, including the limited space in the objective lens pole piece and the possible use of fixed conductive braids to cool the specimen. Consequently, most tilt series are limited to a maximum of ±70°, leading to the presence of a missing wedge in Fourier space. The acquisition of cryo-ET data without a missing wedge, for example using a cylindrical sample geometry, is hence attractive for volumetric analysis of low-symmetry structures such as organelles or vesicles, lysis events, pore formation or filaments for which the missing information cannot be compensated by averaging techniques. Irrespective of the geometry, electron-beam damage to the specimen is an issue and the first images acquired will transfer more high-resolution information than those acquired last. There is also an inherent trade-off between higher sampling in Fourier space and avoiding beam damage to the sample. Finally, the necessity of using a sufficient electron fluence to align the tilt images means that this fluence needs to be fractionated across a small number of images; therefore, the order of data acquisition is also a factor to consider. Here, an *n*-helix tilt scheme is described and simulated which uses overlapping and interleaved tilt series to maximize the use of a pillar geometry, allowing the entire pillar volume to be reconstructed as a single unit. Three related tilt schemes are also evaluated that extend the continuous and classic dose-symmetric tilt schemes for cryo-ET to pillar samples to enable the collection of isotropic information across all spatial frequencies. A fourfold dose-symmetric scheme is proposed which provides a practical compromise between uniform information transfer and complexity of data acquisition.

## Abstract 2

An *n*-helix tilt scheme for cryo-electron tomography of pillar-shaped samples is described and simulated, allowing the entire pillar volume to be reconstructed as a single unit. Three related tilt schemes to enable the collection of the most isotropic information across all spatial frequencies are also evaluated.

## 1. Introduction

Cryo-electron tomography (cryo-ET) is used for three-dimensional reconstruction of beam-sensitive biological samples and enables the structures of biological macromolecules to be determined within their native environment (Frank, 2005 ▸). When used for *in situ* structural biology, cryo-ET is often performed in conjunction with sub-tomogram averaging (STA), in which multiple instances of an object of interest are averaged to obtain a high-resolution reconstructed 3D volume (Ni *et al.*, 2022 ▸). When STA is used in the absence of a strong preferred orientation, and assuming a high number of particles, a reconstruction with isotropic resolution can be obtained from tomograms with a limited tilt range (typically ±60°) due to the random orientations of the particles to be averaged (Wan & Briggs, 2016 ▸; Turoňová *et al.*, 2020 ▸). However, frequently it is not possible to use STA because the objects of interest may not be sufficiently structurally congruent, may exhibit a preferred orientation or may be rare, with only a few instances in each volume. There may also be interest in analysing an entire volume with isotropic resolution which cannot be obtained from a limited tilt range, as for example in studies of vesicles, organelles or bacteria. For example, to calculate the volumes of these classes of objects or when performing an analysis for the presence of holes, such as in a lysis event or for pore-forming proteins, missing-wedge artefacts must be considered (Phillips *et al.*, 2021 ▸). Likewise, missing-wedge artefacts may also cause issues in the analysis of the curvature of the specimen. Having an isotropic angular sampling therefore opens the possibility of more reliable use of artificial intelligence (AI)-based inpainting algorithms as part of standard tomographic reconstruction pipelines (Bellos *et al.*, 2019 ▸). Finally, observing an array of filaments is made more difficult by having a missing wedge, so this must be compensated by finding top views, which may not be straightforward, as the fibres often have a preferred orientation within the cell and this may result in the introduction of unintentional bias.

Electrons interact strongly with matter and, consequently, samples for use in cryo-ET need to be thin (typically <300 nm) to determine structures to high resolution (Henderson, 1995 ▸). A typical cryo-ET workflow involves plunge-freezing or high-pressure freezing cells or tissue samples and then using focused ion beam (FIB) milling to remove material from the sample until it reaches the desired thickness (Marko *et al.*, 2008 ▸; Harapin *et al.*, 2015 ▸; Schaffer *et al.*, 2017 ▸). This typically results in a planar lamella which is thin in the direction of the electron beam but fills the field of view orthogonal to the beam. Planar samples have the inherent limitation that the effective thickness of the sample increases as the sample is tilted such that the effective thickness *D*_eff_ = *D*_zero_/cos(θ), and consequently when a planar lamella is tilted to 60° it will have twice the thickness than that at the zero-tilt position. In thicker samples there is a higher probability of multiple elastic and inelastic events and hence, due to the increase in effective thickness at higher tilt angles, the signal-to-noise ratio (SNR) of the images will be lower and they will contribute less information to the resulting reconstruction. A planar geometry also imposes limits on the range of tilt angles that can be accessed, making it impossible to acquire images across the full 180° tilt range that is required to sample all spatial frequencies. This results in the ‘missing-wedge’ problem in cryo-ET (Radermacher, 1988 ▸; Palmer & Löwe, 2014 ▸; Parkhurst *et al.*, 2021 ▸).

This limitation has created interest in producing cylindrical samples for cryo-ET which would enable on-axis tomography with a full 180° rotation and no missing wedge (Palmer & Löwe, 2014 ▸). This could be achieved by either vitrification of the sample in a pillar geometry (Palmer & Löwe, 2014 ▸; Larabell & Nugent, 2010 ▸; Guo & Larabell, 2019 ▸) or by fabrication of a pillar geometry (Fukuda *et al.*, 2004 ▸; Kawase *et al.*, 2007 ▸; Yaguchi *et al.*, 2008 ▸; Hernández-Saz *et al.*, 2012 ▸). Both approaches have been used in material science and soft X-ray tomography. However, although attempts have been made, the preparation of beam-sensitive biological pillar samples suitable for cryo-ET has proved to be challenging (Palmer & Löwe, 2014 ▸) and has only been achieved using cells in suspension. A more routine and automated method for the creation of pillar-shaped samples is hence required. Recent developments in FIB milling and transmission electron microscope (TEM) instruments have moved closer to realizing this aim. We focus on the instrumentation available to us at the Rosalind Franklin Institute (RFI). The Arctis plasma FIB instrument (Thermo Fisher) in principle enables the production of pillar samples under cryogenic conditions and the manufacturer-modified Krios G4 TEM at the RFI has a tilt stage that can be rotated between ±90°; therefore, the routine production of pillar samples and the collection of TEM data from them is likely to soon become feasible.

Due to the increase in effective thickness at high tilt angles for planar samples, less information is transferred at high tilt angles than at low tilt angles. This has led to the development of the classic dose-symmetric data-acquisition scheme where tilt angles are acquired in order of their absolute magnitude (Hagen *et al.*, 2017 ▸; Turoňová *et al.*, 2020 ▸). This prioritizes data acquisition from tilt angles which are likely to yield the maximum information and is especially useful for data acquisition in STA, where high-resolution information is required but isotropic raw resolution is not necessary (Wan & Briggs, 2016 ▸; Turoňová *et al.*, 2020 ▸). For pillar samples where a full 180° tilt range can be acquired, all tilt angles provide the same amount of information but beam damage will still be limiting; the tilt images acquired earlier in the data acquisition provide higher resolution information than those acquired later. Therefore, the order of image acquisition plays a role in the distribution of information acquired at different spatial frequencies.

In this paper, we describe and simulate an *n*-helix data-acquisition scheme for cryo-ET that can be used to both tilt the sample and shift the sample image to reconstruct large volumes. We also perform an analysis of a set of extended tilt schemes with varied angular sampling for pillar samples of beam-sensitive biological specimens. In particular, we consider imaging without any prior knowledge about the specimen with the aim of providing optimal information transfer over the entire angular range. We describe three families of tilt schemes that can be related through simple rules. These families of tilt schemes are referred to as the spiral, swinging and symmetric schemes. The spiral and swinging schemes extend the continuous data-acquisition scheme and the symmetric scheme extends the classic dose-symmetric scheme. Through analysis of the simulated data sets, we assess the information transfer provided by these schemes as well as their suitability for real-world application by examining the data-acquisition time and fluence gradient between adjacent images. We propose a fourfold dose-symmetric scheme for pillar samples that we believe provides a practical and effective compromise between optimal information transfer, data-acquisition time and data-collection complexity.

## 2. Methods

### 2.1. Properties of data-acquisition schemes for pillars

The properties of an ideal data-acquisition scheme for cryo-ET of pillar samples are as follows.

(i) *Uniform information transfer*: the tilt scheme should produce uniform information transfer at all spatial frequencies to obtain isotropic resolution in reconstructions.

(ii) *Low total accumulated stage tilt*: the tilt scheme should have low total accumulated stage tilt for faster data acquisition.

(iii) *Smooth fluence gradient versus tilt angle*: the tilt scheme should produce a smooth fluence gradient across the angular range with small changes in accumulated fluence between adjacent frames to avoid problems with alignment due to electron-induced sample deformation. This is the ‘jump-at-start’ issue with the traditional bi-directional tilt scheme (Hagen *et al.*, 2017 ▸).

(iv) *Sufficient signal for alignment*: there must be sufficient signal in each tilt image to allow their alignment prior to reconstruction. This limits the extent to which the available electron budget can be fractionated across images.

(v) *High resolution with a large field of view*: in cryo-ET (as in all imaging methods) there is a compromise between the field of view of the data-collection area and the achievable spatial resolution (for a finite detector pixel and array size): to collect higher resolution data, a finer sampling and hence a smaller data-collection area is required. Data acquisition that allows the entire volume to be reconstructed as a single object at high resolution means that rare objects of interest are less likely to be missed and analysis within a larger cellular context can be performed.

(vi) *Uniform fluence distribution versus position*: it is desirable to have a smooth fluence distribution as a function of position across the sample to avoid subjecting some areas of the specimen to more beam damage than others (Peck *et al.*, 2022 ▸). This is particularly important when combined with the desire to analyse the entire pillar as a single volume. The use of overlapping data-collection areas with a square field of view smaller than the circular beam profile implies that electrons outside the data-collection area cause beam damage to the sample but will not provide useful information. Tiling strategies that ensure a uniform fluence distribution can ensure a more spatially uniform transfer of high-resolution information.

### 2.2. Angular sampling

In practice, the cross sections of pillar samples created by FIB milling may be slightly elliptical rather than being perfectly circular, although for the purposes of this analysis we assume that the pillar has a circular cross section. Such pillar samples will have the same effective thickness at all tilt angles, meaning that there is no angular dependence to the information transfer and beam damage to the sample will accumulate over the course of data acquisition, with the last images in the tilt series transferring less high-resolution information than the first images. Therefore, the order of image acquisition is important. There are *N*! possible angular sampling schemes for a set of *N* tilt angles. Many of these orderings will have no practical use; however, it is instructive to consider whether there is any benefit to collecting images in any order other than in the typical continuous or classic dose-symmetric schemes. To investigate different tilt schemes, we define three related families of extended tilt schemes suitable for pillar samples with a ±90° tilt range which can be used in conjunction with the *n*-helix tilt scheme described in Section 2.3.

**Figure 1:**
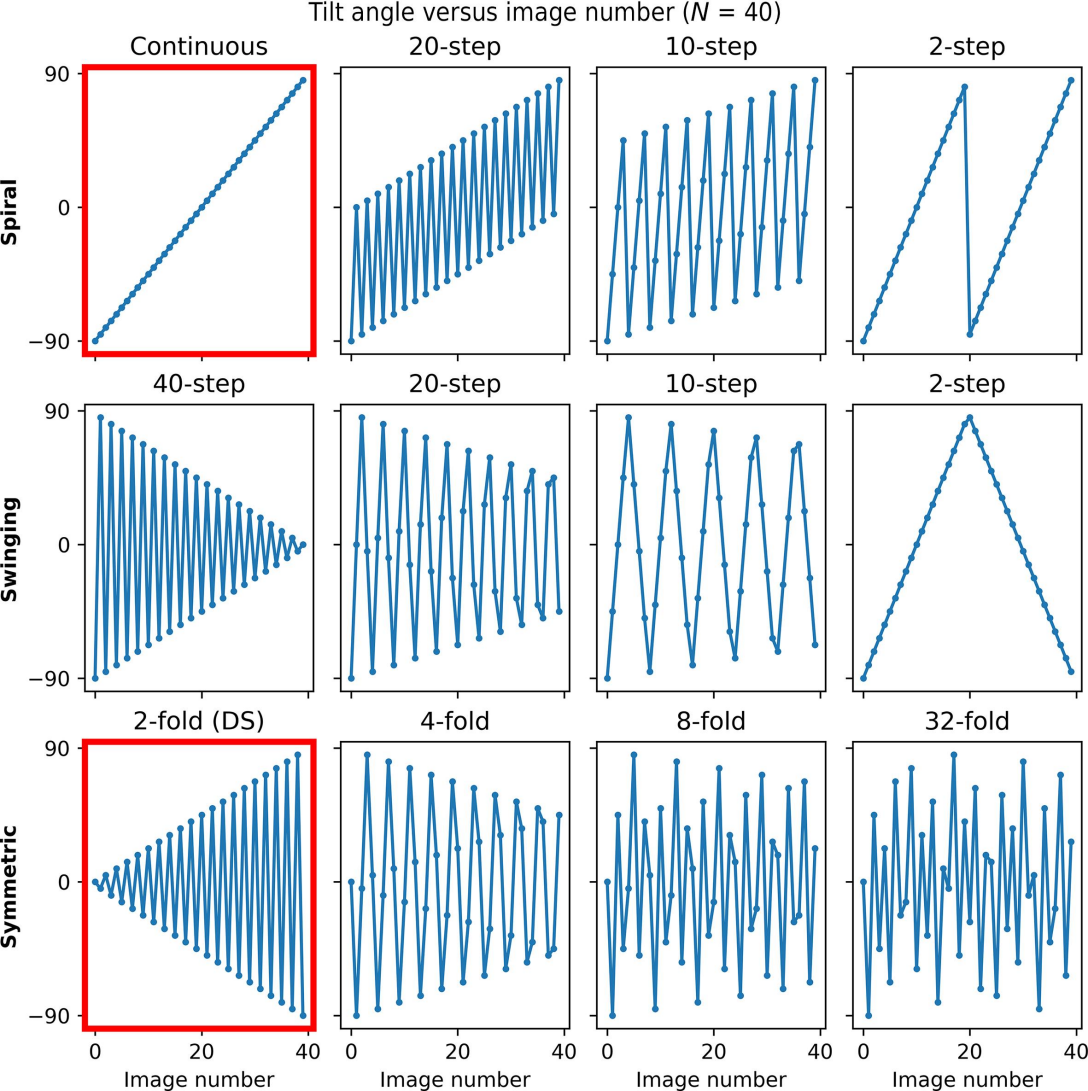
Tilt angle as a function of image number for the spiral (top), swinging (middle) and symmetric (bottom) schemes. Four tilt schemes from each family are shown with symmetry increasing from left to right. The continuous and classic dose-symmetric (DS) schemes are highlighted in the plots outlined in red.

*Spiral*. The continuous tilt scheme is extended as shown in the top row of Fig. 1 ▸. The list of angles is ‘de-interleaved’ into a set of sublists by stepping a number of angles *M*, where *M* ≥ 1, returning to the start position, and collecting *M* tilt series moving from negative to positive angles for each consecutive tilt series. For example, consider a tilt range of ±90° and a step size of 2°. For *M* = 1, this simply produces the continuous tilt scheme moving from −90° to +90° in order. For *M* = 2, this produces the two interleaved tilt series (–90, −86, …, −2, +2, …, +82, +86) followed by (−88, −84, …, 0, …, +84, +88).

*Swinging*. The continuous scheme is extended as shown in the middle row of Fig. 1 ▸. As for the spiral scheme, the set of angles is ‘de-interleaved’ into a set of sub-lists by stepping a number of angles *M*, where *M* ≥ 1, and collecting *M* tilt series. However, in the swinging scheme the direction of collection is alternated, first going from negative to positive and alternately going from positive to negative angles for each consecutive tilt series. For example, again consider a tilt range of ±90° and a step size of 2°. For *M* = 2, this produces the two interleaved tilt series (−90, −86, …, −2, +2, …, +82, +86) followed by (+88, +84, …, 0, …, −84, −88).

*Symmetric*. The classic dose-symmetric scheme is extended as shown in the bottom row of Fig. 1 ▸ by recursively dividing the set of angles by two at the middle of the ordered list, reversing the second subset of angles and then interleaving the resulting subsets in groups of *M* angles to produce 2^*M*+1^-fold dose-symmetric schemes which optimally spread the dose across the angular range. For example, again considering a tilt range of ±90° and a step size of 2°, for *M* = 1 this produces the following fourfold dose-symmetric scheme (0, −90, −2, +88, 2, −88, −4, +86, …) which samples orthogonal directions uniformly.

The order of tilt-image acquisition for the three families of tilt schemes is illustrated in Fig. 1 ▸, which shows the tilt angle as a function of image number for a tilt scheme consisting of 40 equally spaced images over a range of ±90°. The continuous scheme and classic dose-symmetric (DS) scheme are highlighted in red. The continuous scheme is shown as a straight line with angles collected from −90° to +90° in ascending order. The classic dose-symmetric scheme defines a triangle from 0° with larger swings at the end of the tilt-series acquisition. For each of the three families of tilt schemes, the figure columns group the tilt schemes with similar approximate symmetry. It is immediately obvious that some tilt schemes are related: the 40-step swinging scheme is the reverse of the classic (twofold) dose-symmetric scheme. The 20-step swinging scheme is comparable to the fourfold dose-symmetric scheme. The higher order symmetric schemes visit angles in a specific order; however, the tilt-stage movement is more complex. More information about the tilt-scheme construction can be found in Section S1. The effective symmetry of the tilt schemes is further illustrated in Supplementary Fig. S4, which shows a schematic of the image-acquisition order for each tilt scheme.

### 2.3. *n*-Helix data acquisition

The acquisition of high-resolution imaging data in cryo-ET typically requires the use of a high magnification to sample the features of interest in the specimen. This in turn limits the size of the field of view that can be imaged. To analyse larger cellular volumes, it is necessary to image and reconstruct the specimen with a larger field of view. Montage tomography enables the reconstruction of large volumes/areas without sacrificing resolution (Mastronarde, 2005 ▸; Peck *et al.*, 2022 ▸; Yang *et al.*, 2023 ▸). In this method, the image-acquisition area is tiled across the specimen using either beam shifts or stage shifts, multiple tilt series are acquired and the resulting tomograms are computationally stitched. A key issue for montage tomography of beam-sensitive biological samples is ensuring an optimal distribution of electron fluence across the sample, since some areas of the sample will inevitably receive a higher fluence due to the overlapping regions necessary for stitching together the volume and the circular and rectangular or square shape of the electron beam and detector, respectively. To mitigate this issue, the use of square beam profiles has recently been proposed (Chua *et al.*, 2023 ▸); however, circular beam profiles are currently more commonly available in cryo-ET. For circular beam profiles, optimal tiling methods have been proposed for planar samples involving global rotations and translations to reduce the variance in the fluence across the sample, thereby enabling high-resolution large field-of-view tomographic data collection (Peck *et al.*, 2022 ▸).

**Figure 2:**
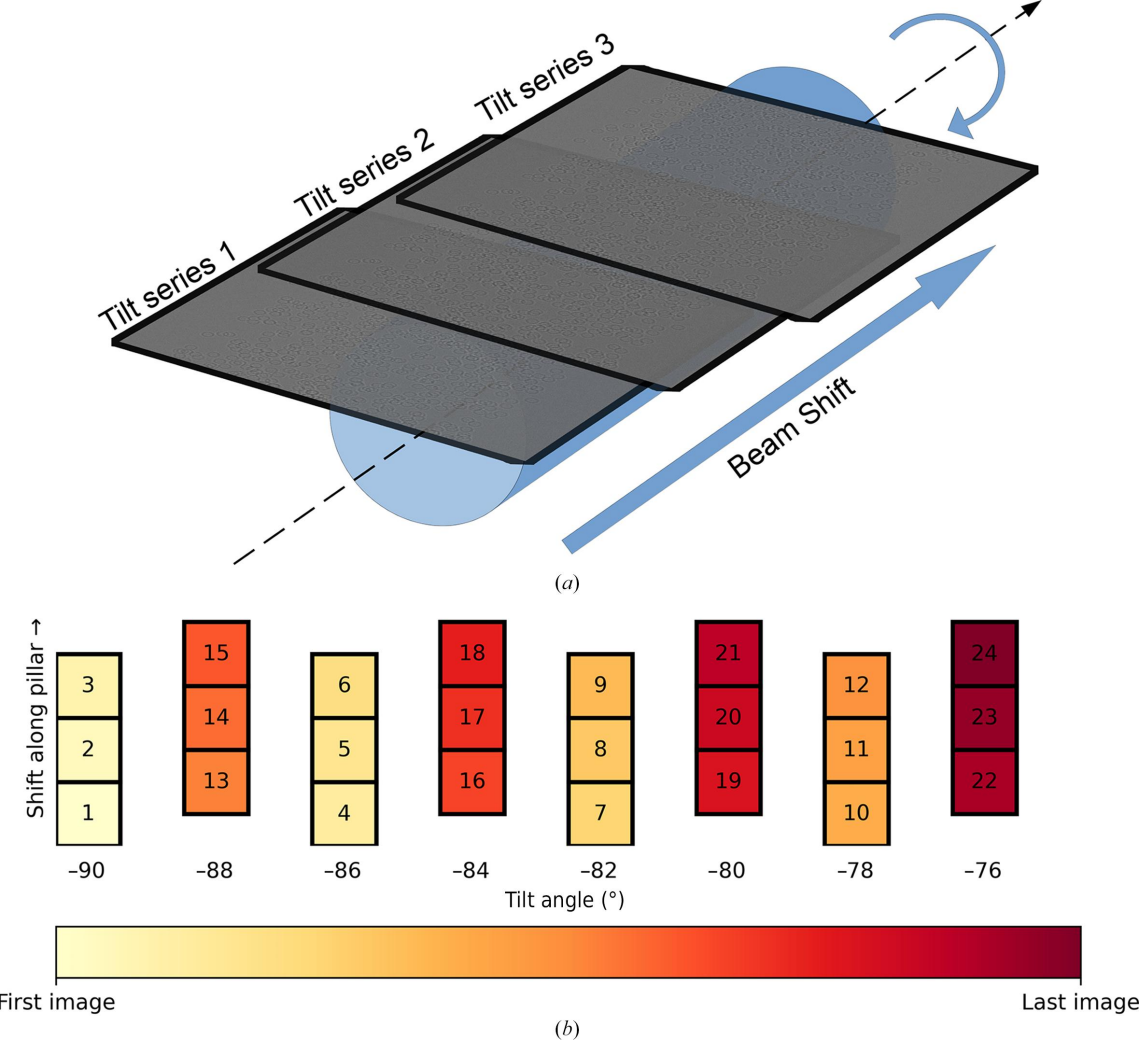
Schematic illustrating the *n*-helix tilt scheme for *n* = 2 overlapping tilt series with 50% overlap between sweeps. (*a*) The overlapping data-collection areas as the beam is shifted along the axis of the pillar sample. (*b*) The order of image acquisition between −90° and −76° is illustrated by image number and colour as light (first image) to dark (last image). The beam is shifted along the pillar to acquire tilt images at each angle before tilting the stage to the next angle. In this example, the beam is then shifted again along the sample to overlap the previous acquisition area by 50% and the intermediate angles which were skipped in the first pass are then acquired at each beam-shift position.

To make the best use of a pillar geometry for acquiring tomograms of large sample volumes, we propose an *n*-helix data-acquisition scheme, as illustrated in Fig. 2 ▸. In this scheme, the desired angular sampling is first defined and the total electron fluence is then divided amongst *n* tilt series which are interleaved, and the beam is shifted along the rotation axis to enable acquisition and reconstruction along the length of the sample. As the beam is shifted along the pillar, the tilt series are overlapped to allow the full volume to be ‘stitched’ whilst maintaining a desired total fluence in any one area of the sample. The tilt series are interleaved such that overlapping regions are sampled at intermediate tilt angles. Stitching together a large volume through the tiling of data-acquisition areas in montage tomography necessarily requires overlapping of the data-collection areas. In the case of pillar data acquisition, this means that the ends of the pillar will receive a lower electron fluence than the central region of the pillar data-acquisition area. Depending on the data-acquisition parameters used, there will hence be some variance in the total accumulated fluence across the sample; strategies to minimize this are discussed in more detail in Section 3.6.

Traditionally, in cryo-ET, tracking and focusing needs to be performed during data acquisition for each tilt series collected. Recent developments in data-acquisition software emphasize the use of beam shifts and a geometric model of the sample, tilt axis and beam profile to collect multiple tilt series for a single tracking and focusing area (Eisenstein *et al.*, 2023 ▸). This method has several benefits in that it requires a smaller area of the sample for tracking and focusing, so more of the specimen can be used to collect structural data. This also speeds up data acquisition since less time is spent tracking and multiple tilt series can be collected for a single tilt-stage movement. The problem with this method when applied to planar lamellae is in ensuring that the sample geometry is known to enable good prediction for off-tilt-axis positions on the lamellae. For pillar samples, this task is simplified by the fact that a model for a line is needed rather than a model for a plane. Therefore, one practical implementation for the *n*-helix data-acquisition scheme is to track in a single location, at the base of the pillar or in an area with sufficient features to align the *x* and y directions, and then to utilize beam shifts along the pillar to acquire images at all the desired locations for a given tilt angle, whilst compensating for the beam-tilt-induced aberrations caused by the beam shift.

### 2.4. Simulation of cryo-ET images

Tilt series of images were simulated using the *Parakeet* software package (Parkhurst *et al.*, 2021 ▸) which uses the MULTEM library (Lobato & Van Dyck, 2015 ▸) to perform multislice simulations (Cowley & Moodie, 1957 ▸; Goodman & Moodie, 1974 ▸). The simulations were performed using a beam-damage model in *Parakeet* where a static *B* factor increases linearly with the number of incident electrons as *B* = 8π^2^ *D*_E_ *S*_E_, where *D*_E_ is the total number of accumulated incident electrons for a given image and *S*_E_ is a sensitivity coefficient which typically takes values between 0.02 and 0.08 Å^4^/e^−^ (Parkhurst *et al.*, 2021 ▸). This *B* factor is applied to the atomic potential of the sample during the multislice calculation and serves to progressively blur the atomic potential for each subsequent image in a tilt-series simulation. In this way, as the number of images acquired increases and, therefore, the number of electrons incident on the sample increases, the high-resolution features in the sample are reduced. This simple model accounts for the initial stages of the beam-damage process and approximates the effects of the limit of information transfer in the samples. This model is also consistent with the beam-damage model used in the Bayesian polishing algorithm in *RELION* (Zivanov *et al.*, 2019 ▸). However, it does not consider the specific mechanisms involved in the damage process and is less well suited to modelling the latter stages of the damage process such as bubble formation. It should also be noted that the damage rate was calibrated from protein data; the damage rates for membranes, RNA and DNA may be different. The atomic *B* factors were assumed to be zero before radiation damage was applied. Incorporating the beam-damage model into these simulations is important as it is necessary for the order of the images to have an effect on the data quality. Finally, the effect of the ice in the sample was modelled using a Gaussian random field (GRF) model of the amorphous ice potential, which allows large volumes of ice to be simulated efficiently (Parkhurst *et al.*, 2024 ▸). Using *Parakeet*, a set of samples containing 1000 ribosomes embedded in pillars with diameters of 300 nm were generated. Tilt-series simulations were then performed using this model for the different tilt schemes. A full set of simulation parameters is given in Table 1 ▸.

### 2.5. Assessment of reconstruction quality

To assess the quality of the reconstructions produced by the different tilt schemes, tomograms were reconstructed using a 3D contrast transfer function (CTF)-corrected weighted back-projection (WBP) algorithm (Turoňová *et al.*, 2017 ▸). The particles were then extracted from the reconstructed volumes and split into two sets. The two half sets of particles were then aligned and averaged, and the reconstruction quality was assessed by computing the Fourier shell correlation (FSC) between the two half sets (Harauz & van Heel, 1985 ▸). The resolution was determined using the point at which the FSC first drops below a value of 0.143 (Rosenthal & Henderson, 2003 ▸). This process was performed for different numbers of particles to quantify the resolution as a function of the number of particles. To assess the anisotropy of the information transfer, the FSC was also analysed in Fourier space planes to highlight the directions in which the various tilt schemes transfer the most information.

## 3. Results and discussion

### 3.1. Alignment of images in pillar and planar samples

**Figure 3:**
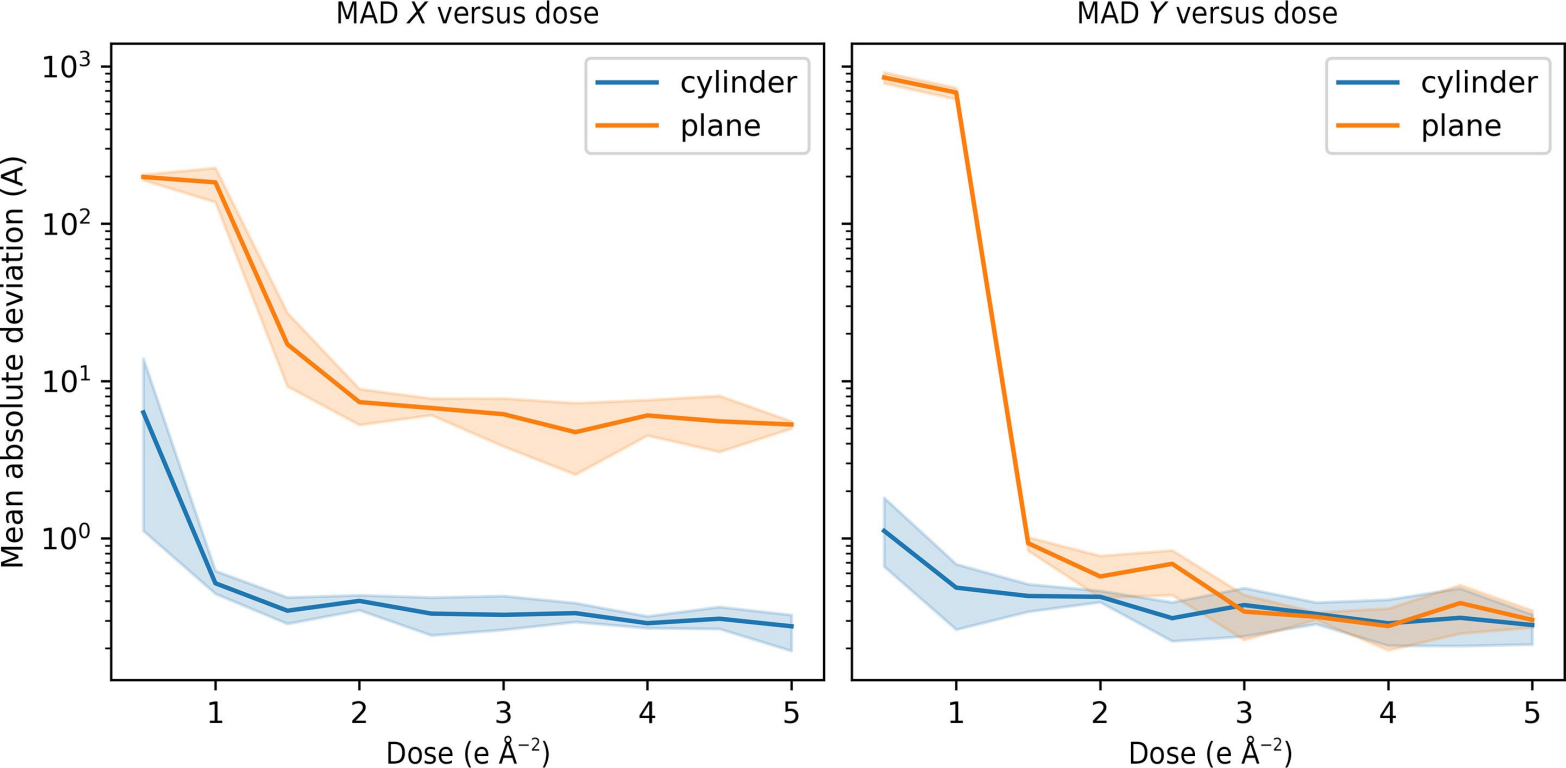
MAD between the known and estimated image offsets as a function of fluence in the *X* direction (left) and the *Y* direction (right). The plots show the alignment errors for both planar (orange) and pillar (blue) samples. The shaded area shows the fifth and 95th percentiles of the alignment errors.

A set of synthetic cryo-ET data sets were simulated for both planar and pillar-shaped samples with an electron fluence ranging from 0.5 to 5 e^−^ Å^−2^ per tilt image and a pixel size of 1 Å. For each fluence, tilt series with zero, small (σ = 10 Å) and large (σ = 50 Å) random alignment errors were simulated. To highlight the differences between planar and cylindrical samples, for this analysis the tilt series were simulated using a continuous acquisition scheme. These tilt series were then aligned using a projection-matching cross-correlation-based algorithm (Gürsoy *et al.*, 2017 ▸). In general, this alignment solution is not unique and depends on the estimated centre of rotation. Therefore, to aid comparison with the known ground-truth positional offset, a model was fitted to the raw *X* residuals between the observed and expected offsets, *r*_*x*_, using least squares to minimize 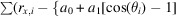 + 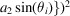 , where *a*_0_, *a*_1_ and *a*_2_ are the fitted parameters and θ is the tilt angle. The corrected *X* residuals were then used in the analysis of the alignment errors. The mean absolute difference (MAD) between the known offsets needed to align the tilt images and the estimated offsets was then calculated. Fig. 3 ▸ shows the alignment error expressed as the MAD as a function of fluence per tilt image in the *X* direction (orthogonal to the rotation axis) and the *Y* direction (parallel to the rotation axis). In the *X* direction, the alignment of the pillar samples is more than an order of magnitude better than the alignment of the planar samples across the entire range of fluence considered. The alignment errors increase at low fluence due to the lack of signal in the images; however, the fluence at which the alignment errors increase substantially is lower for pillars than it is for planar samples. For planar samples the MAD in *X* is greater than 10 Å for a fluence of 1.5 e^−^ Å^−2^, but for pillar samples the MAD in *X* is still lower than 10 Å for a fluence of 0.5 e^−^ Å^−2^. This is likely to be due to the presence of the pillar edge, which generates external contrast between the sample and vacuum. In comparison, the alignment of planar samples which extend beyond the field of the view of the images depends entirely on the internal contrast of the sample. As a result, the electron budget for individual images can be decreased for pillar relative to planar samples whilst still enabling alignment of the tilt images. This enables the fluence to be fractionated over a larger number of images, which in turn allows a greater number of spatial frequencies to be sampled relative to that possible for planar samples. The alignment in the *Y* direction is similar for both planar and pillar samples across most of the range of fluence considered; however, for planar samples the poor alignment at low fluence also causes a large jump in the magnitude of the *Y* alignment errors at low fluence; the magnitude of the *Y* alignment errors for the pillar samples remains low, with a MAD below 1 Å, in comparison. It should be noted that the images used to evaluate the alignment were simulated without gold fiducials. Alignment is expected to be better determined with a lower MAD for both pillar and planar samples if an accurate fiducial model can be constructed.

**Figure 4:**
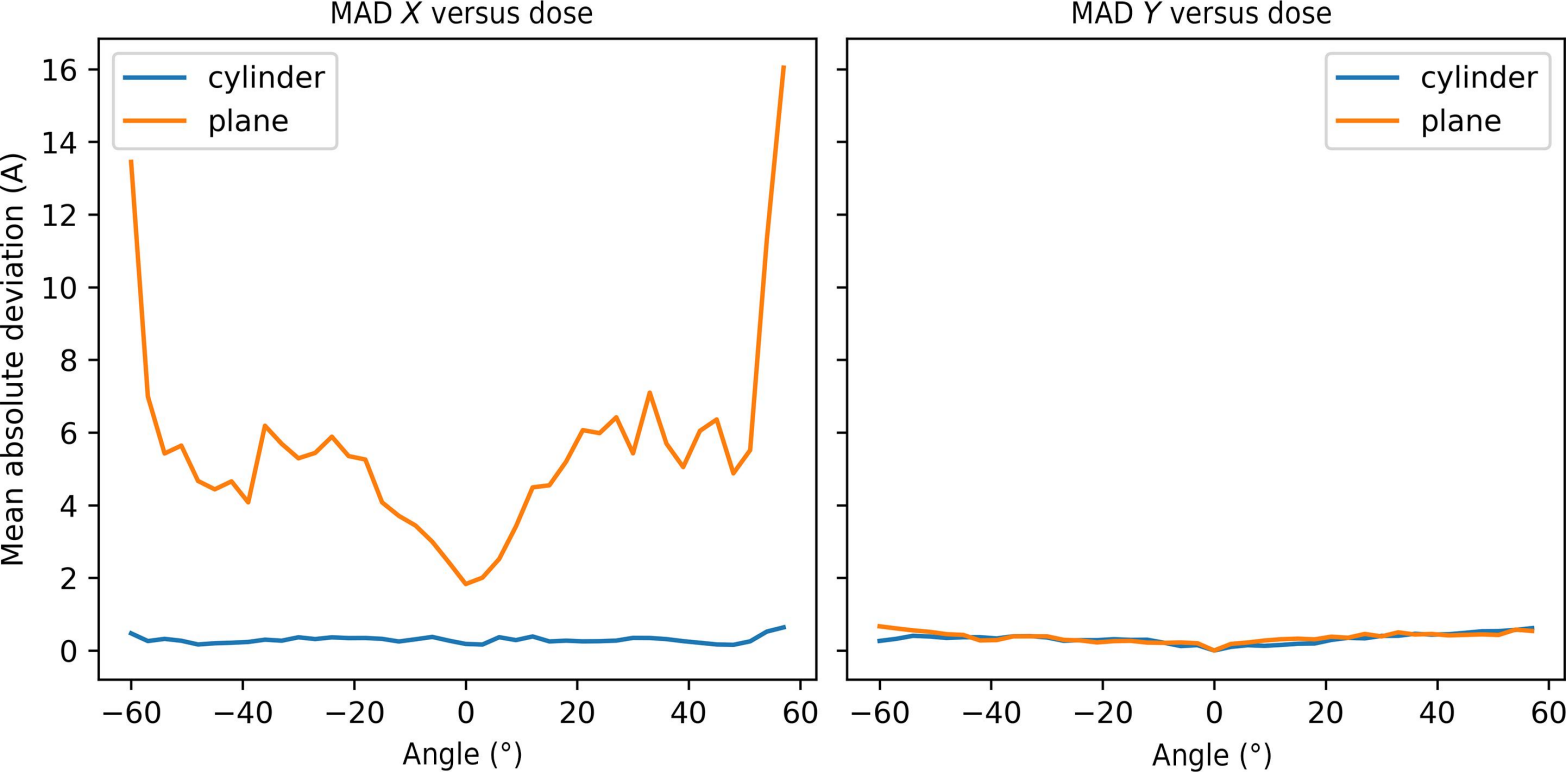
MAD between the known and estimated image offsets as a function of tilt angle in the *X* direction (left) and the *Y* direction (right). The plots show the alignment errors for both planar (orange) and pillar (blue) samples.

It is also instructive to observe the magnitude of the alignment errors as a function of tilt angle, as shown in Fig. 4 ▸. Here, the absolute deviations between the fitted models and the expected offsets were averaged for each tilt angle across all data sets with a fluence of ≥2.5 e^−^ Å^−2^ per image. The MAD was then plotted as a function of tilt angle. It can be seen that the alignment of the planar sample becomes progressively worse at high tilt angles above ±20°, which accounts for the poor overall MAD of the planar samples in Fig. 3 ▸. The alignment of the pillar samples does not vary significantly as a function of tilt angle, although there is a marginal increase at high tilt angles due to the accumulation of errors, since images at high tilt angles are aligned to reference images at lower tilt angles.

### 3.2. Information transfer as a function of angular sampling

The angular distribution of the information transfer depends on several factors, including the total incident fluence, the beam sensitivity of the specimen, the fluence and flux for a particular image, the total number of tilt images in the data acquisition and the order of image acquisition. In the following analysis, we assume that each image receives the same number of incident electrons, and that the *B* factor increases linearly as a function of the number of incident electrons such that *B* = 8π^2^ *D*_E_ *S*_E_, where *D*_E_ is the fluence and *S*_E_ is a sensitivity coefficient. The total fluence across the data sets is assumed to be 140 e^−^ Å^−2^, which is typical for that used in cryo-ET, and this is assumed to be evenly distributed across the 40 images in the tilt series. In the following analysis, a value of *S*_E_ = 0.04 Å^4^/e^−^ was used.

**Figure 5:**
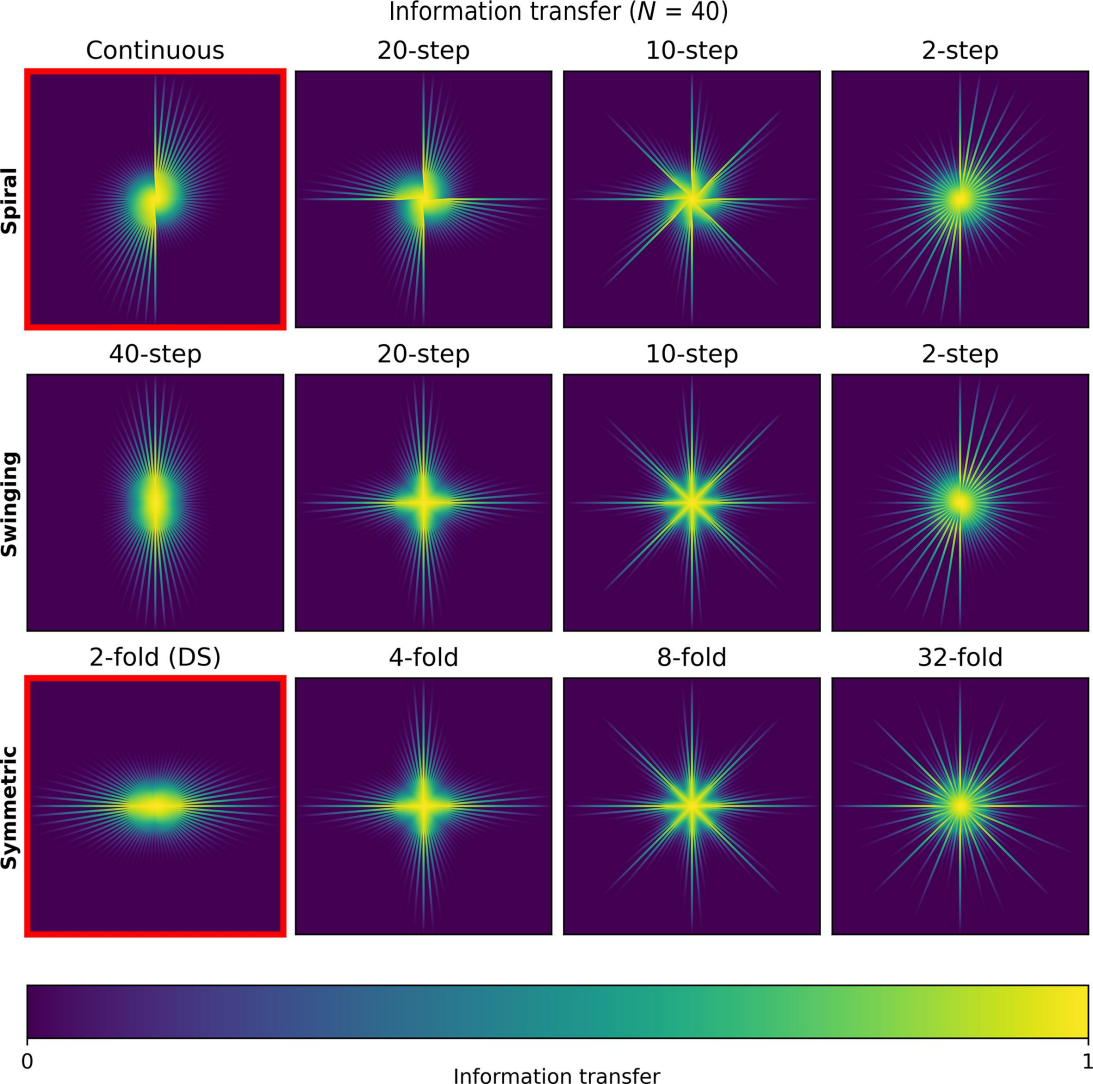
Information transfer in the reciprocal *X*Z plane in Fourier space for the spiral (top), swinging (middle) and symmetric (bottom) schemes. Four tilt schemes from each family are shown with symmetry increasing from left to right. The continuous and classic dose-symmetric (DS) schemes are outlined in red. The information transfer is the envelope function on the amplitudes of the Fourier coefficients produced by a given *B* factor. High information transfer is shown in yellow and low information transfer is shown in blue, as illustrated by the colour bar. In each case, the Nyquist limit is 2 Å.

The information transfer in Fourier space is shown in Fig. 5 ▸. The plots show the centred damping envelope resulting from the blurring due to the beam damage as given by the relative *B* factor. Spatial frequencies in between the tilt images are not sampled. For zero damage all spatial frequencies in a given direction will be sampled, but for a damaged specimen only low spatial frequencies in a given direction will be sampled. Hence, the distribution of the information transfer varies considerably between the different tilt schemes. It should be noted that in all of these plots the total integrated information transfer is the same. Choosing to collect information in one direction is equivalent to choosing not to collect information in another direction. The plots highlight the same symmetry as seen in the polar *B*-factor plots in Supplementary Fig. S5. The spiral family produces approximate rotational symmetry for large step sizes. The swinging scheme produces approximate rotational and mirror symmetry in the information transfer for large step sizes. The most uniform information transfer is given by the symmetric schemes, which distribute the information optimally for a specific symmetry. Increasing the symmetry results in an increasing uniformity of the spread of the fluence across the angular range. However, the maximum symmetry for the data-acquisition scheme is limited by the total number of tilt images used. The classic dose-symmetric scheme as highlighted in the figure prioritizes information transfer along a single direction in the reciprocal *XZ* plane in Fourier space.

### 3.3. Anisotropy of information transfer

**Figure 6:**
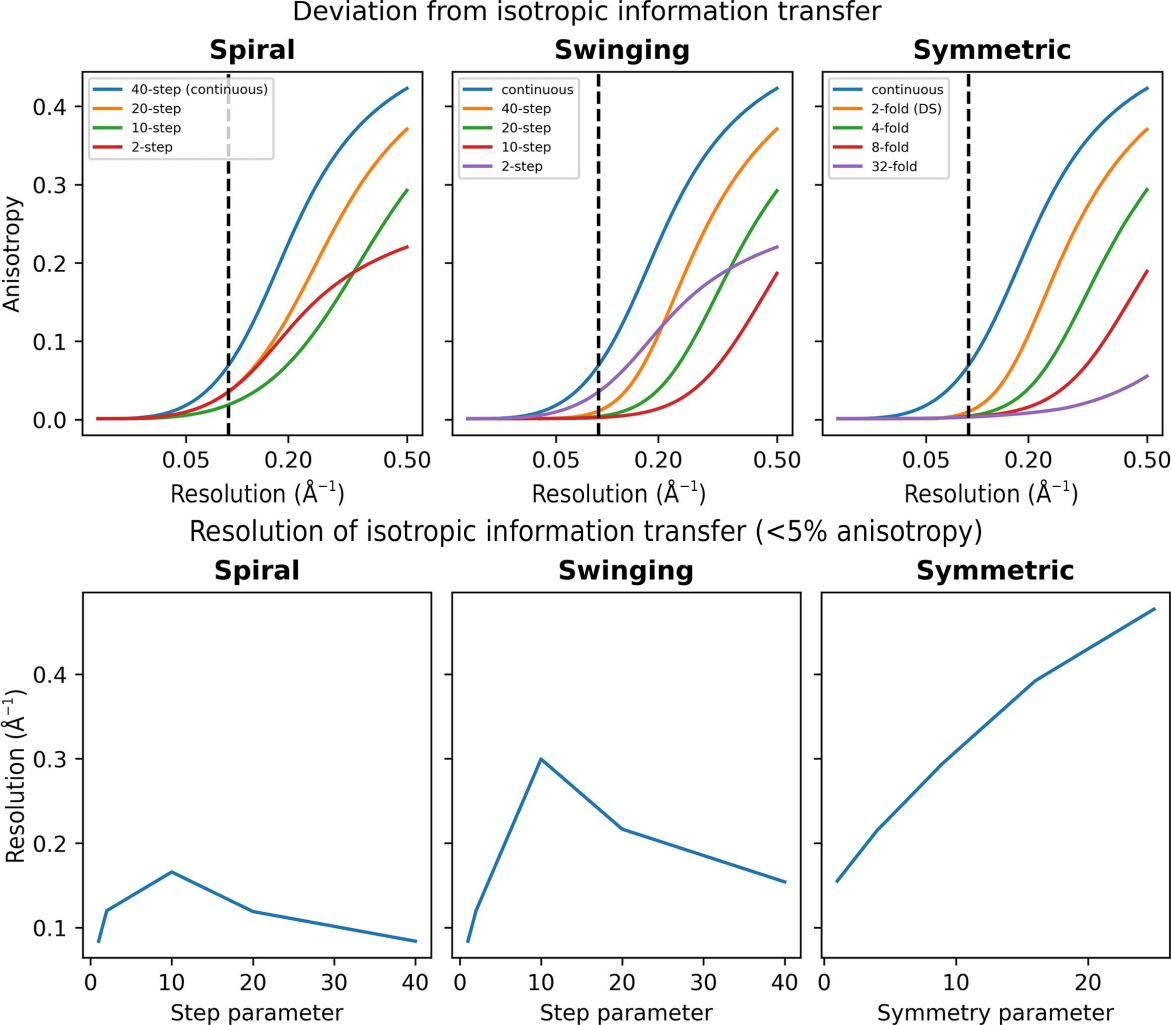
Top row: anisotropy of information transfer for the spiral (left), swinging (middle) and symmetric (right) tilt schemes. The continuous scheme is shown in blue for each panel and the classic dose-symmetric (DS) scheme is shown in the right panel. The black dashed vertical line at a resolution of 10 Å highlights the resolution range at which the pillar geometry is likely to provide a major advantage over a planar geometry. Bottom row: the resolution at which the information transfer is approximately isotropic (with less than 5% anisotropy) for the spiral (left), swinging (middle) and symmetric (right) tilt schemes.

The anisotropy of the information transfer as a function of resolution is shown in Fig. 6 ▸. Here, the anisotropy at a given resolution is defined using the Kolmogorov–Smirnoff test statistic (Kolmogorov, 1933 ▸; Smirnov, 1948 ▸) assuming a uniform directional distribution. This has a value of zero if the information transfer is completely uniform and a value of one if the distribution of information transfer is unidirectional. In general, this quantity increases as a function of resolution for all tilt schemes. For beam-sensitive samples, the continuous scheme has the most anisotropic information transfer, as most of the information is transferred in a single direction. For the spiral scheme, for the parameters used here, the two-step scheme has the lowest anisotropic information transfer at high resolutions above 3 Å, but the ten-step scheme is better at intermediate resolutions up to around 3 Å. The swinging scheme follows a similar pattern, with lower step sizes yielding better isotropic information transfer up to a point, with the ten-step scheme, with approximate eightfold symmetry, having the most uniform information transfer in this example. For the symmetric schemes, the higher order symmetry schemes have more isotropic information transfer across all spatial frequencies, although the transfer decreases with each increase in symmetry, suggesting diminishing returns in using higher symmetry schemes.

### 3.4. Accumulated stage tilt

**Figure 7:**
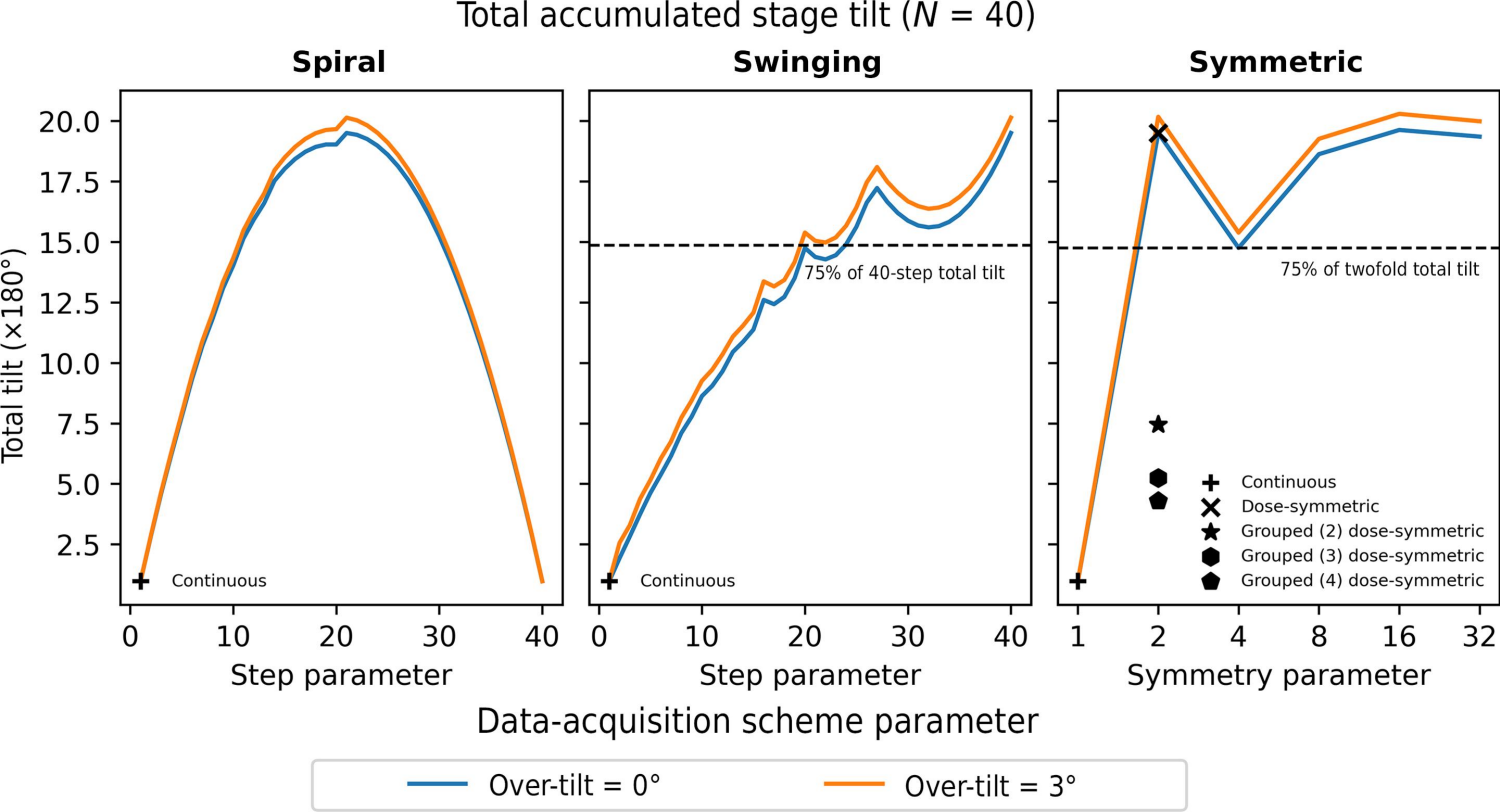
Total accumulated stage tilt for spiral (left), swinging (middle) and symmetric (right) tilt schemes. The total accumulated stage tilt is shown for no over-tilt and an over-tilt of 3°. The continuous, classic dose-symmetric and grouped dose-symmetric tilt schemes are highlighted in the plots where applicable.

The total accumulated stage tilt of a data-acquisition scheme primarily affects the time required to acquire the tilt series. Data-acquisition schemes with larger total accumulated stage tilt will also take longer to execute, potentially limiting the throughput. The continuous tilt scheme has the lowest possible total accumulated stage tilt, given that the stage tilts continuously from −90° to +90° with no back-tracking. In contrast, the classic (twofold) dose-symmetric scheme has the largest possible total accumulated stage tilt. This is evident from a consideration of the classic dose-symmetric scheme in reverse for a given set of angles; the last stage movement requires the largest change in angle, while removing these two positions the second-to-last stage movement then has the next largest change in angle. When using the classic dose-symmetric scheme for planar samples this issue is often mitigated by using a grouped symmetric acquisition scheme, where two or more adjacent tilt images are collected before reversing the direction of acquisition, thereby reducing the total accumulated stage tilt. As shown in Fig. 7 ▸, for spiral tilt schemes the total accumulated stage tilt increases with step size until the step size reaches half the number of tilt images. The accumulated stage tilt then decreases. For the swinging schemes, the total accumulated stage tilt increases depending on the angular sampling as the step size increases. As an example, for a step size of 40 equal to the number of tilt images, the tilt scheme is the reverse of the classic dose-symmetric scheme, and this has the largest possible total accumulated stage tilt. For the symmetric family of tilt schemes, the fourfold scheme has a lower total accumulated stage tilt than the classic (twofold) dose-symmetric scheme, with 75% of required tilt compared with the twofold scheme. The higher symmetry schemes show an increase in the total accumulated tilt. The ten-step swinging scheme, which has eightfold symmetry, requires much lower total accumulated tilt than the true eightfold symmetric scheme. Typically, in tilt schemes which involve back-tracking, such as the classic dose-symmetric scheme, it is desirable to approach the target angle from the same direction to avoid mechanical stage ‘backlash’ (Turoňová *et al.*, 2020 ▸). Fig. 7 ▸ also shows the total accumulated stage tilt including a 3° ‘over-tilt’ to ensure that the target angle is always approached from the negative direction. With the over-tilt, the total accumulated stage tilt is higher, but the same arguments apply as for cases with no over-tilt.

### 3.5. Accumulated fluence jump at angle

**Figure 8:**
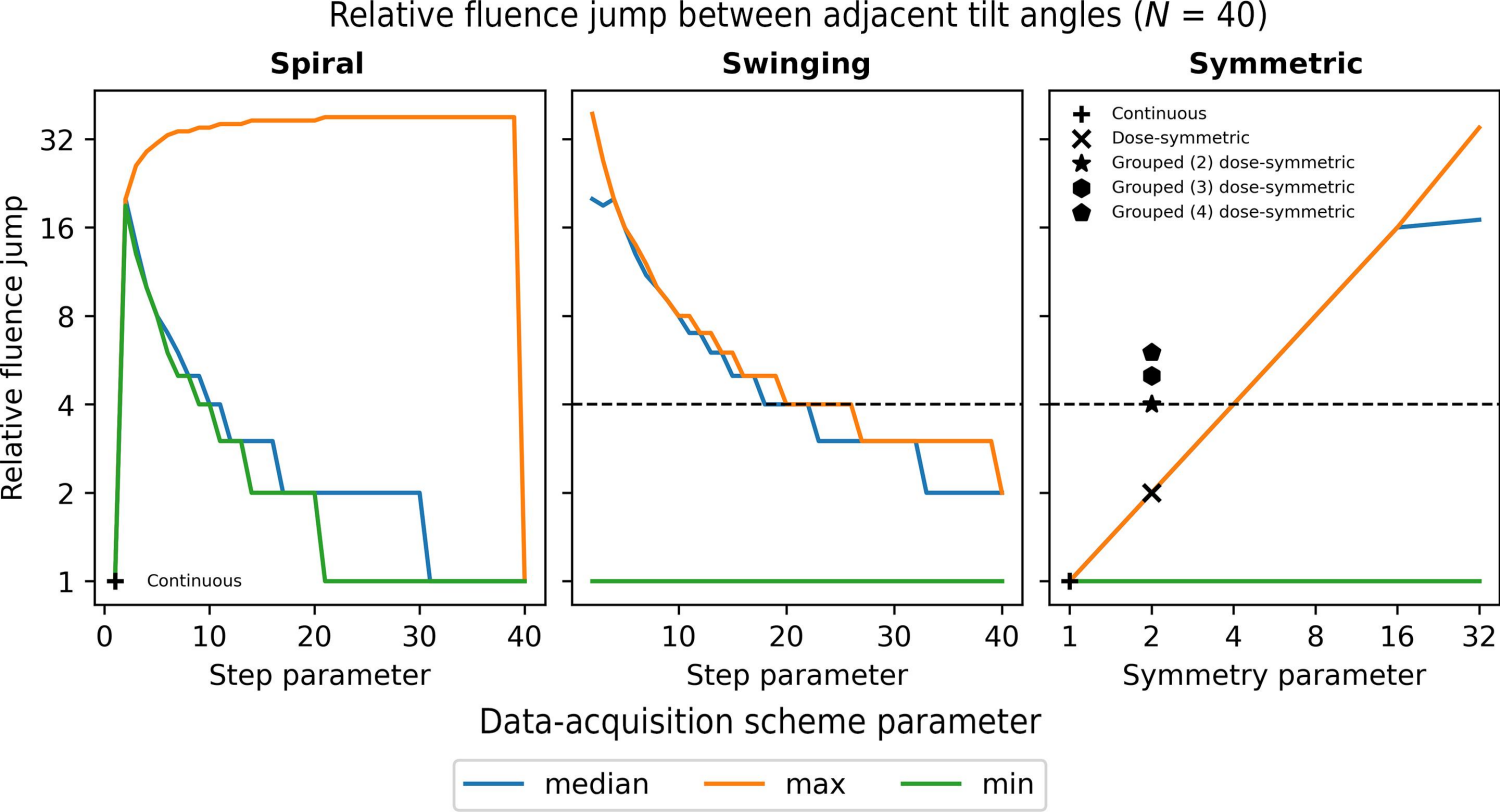
Minimum, maximum and median relative fluence jump between adjacent tilt angles for the spiral (left), swinging (middle) and symmetric (right) tilt schemes. The continuous, classic dose-symmetric and grouped dose-symmetric tilt schemes are highlighted in the plots. The horizontal dashed line in the swinging and symmetric scheme plots shows the fluence jump for the fourfold symmetric schemes.

If images at adjacent tilt angles have a major difference in accumulated fluence, there may be problems with alignment due to electron-induced sample deformation (Hagen *et al.*, 2017 ▸). This is known as the ‘jump-at-start’ issue for the traditional bi-directional tilt scheme where, starting from zero tilt, the negative tilt branch is collected followed by the positive tilt branch (Hagen *et al.*, 2017 ▸). Additionally, charging of the sample in the electron beam also results in beam-induced motion and image distortion (Zhang *et al.*, 2023 ▸). One of the reasons that the classic dose-symmetric scheme is preferred for planar samples is that the fluence varies smoothly between adjacent images. If each image receives the same fluence, then we define the fluence jump as the step in accumulated fluence between adjacent images. The continuous tilt scheme has a fluence jump of 1 and the classic dose-symmetric scheme has a maximum fluence jump of 2 between adjacent images. Fig. 8 ▸ shows the median, maximum and minimum fluence jumps for the spiral, swinging and symmetric tilt scheme families. For the symmetric schemes, the maximum fluence jump between adjacent images is equal to the symmetry of the scheme. The spiral schemes (except the continuous scheme) all have a large fluence jump between adjacent images. However, the swinging schemes with large step sizes have smaller fluence jumps consistent with the approximate symmetry of the schemes. In general, the trade-off to having a more uniform distribution of information transfer is to have a larger fluence gradient between adjacent images.

In many cases, it is necessary to remove tilt images from the data set due to the presence of contamination, due to Bragg diffraction from crystalline material or due to poor tracking during data acquisition. Ideally, tilt schemes need to be robust to the removal of such images. The robustness will be proportional to the relative fluence jump between adjacent images in that if an image is removed from the tilt series then the maximum fluence jump between adjacent images will double. Therefore, robustness to the removal of images is another factor that makes it desirable to have a lower relative fluence jump between adjacent tilt images.

### 3.6. Fluence distribution as a function of position

To enable the reconstruction of an entire pillar as a single object, the *n*-helix data-acquisition strategy collects images from overlapping regions on the sample which are subsequently stitched together during reconstruction. The number of overlapping regions is allowed to vary according to the value of *n*. For example, the one-helix strategy has no overlaps in the data-acquisition area; each sweep position is shifted by a whole field of view. For the two-helix strategy, images at every second angle are shifted by 1/2 of the field of view to allow 50% overlap in the data-acquisition position, thereby allowing the fields of view to be stitched together. For the four-helix and the eight-helix strategies, images at adjacent tilt angles are shifted by 1/4 and 1/8 of the field of view, respectively. Hence, the use of different *n*-helices will result in different distributions of fluence across the sample.

**Figure 9:**
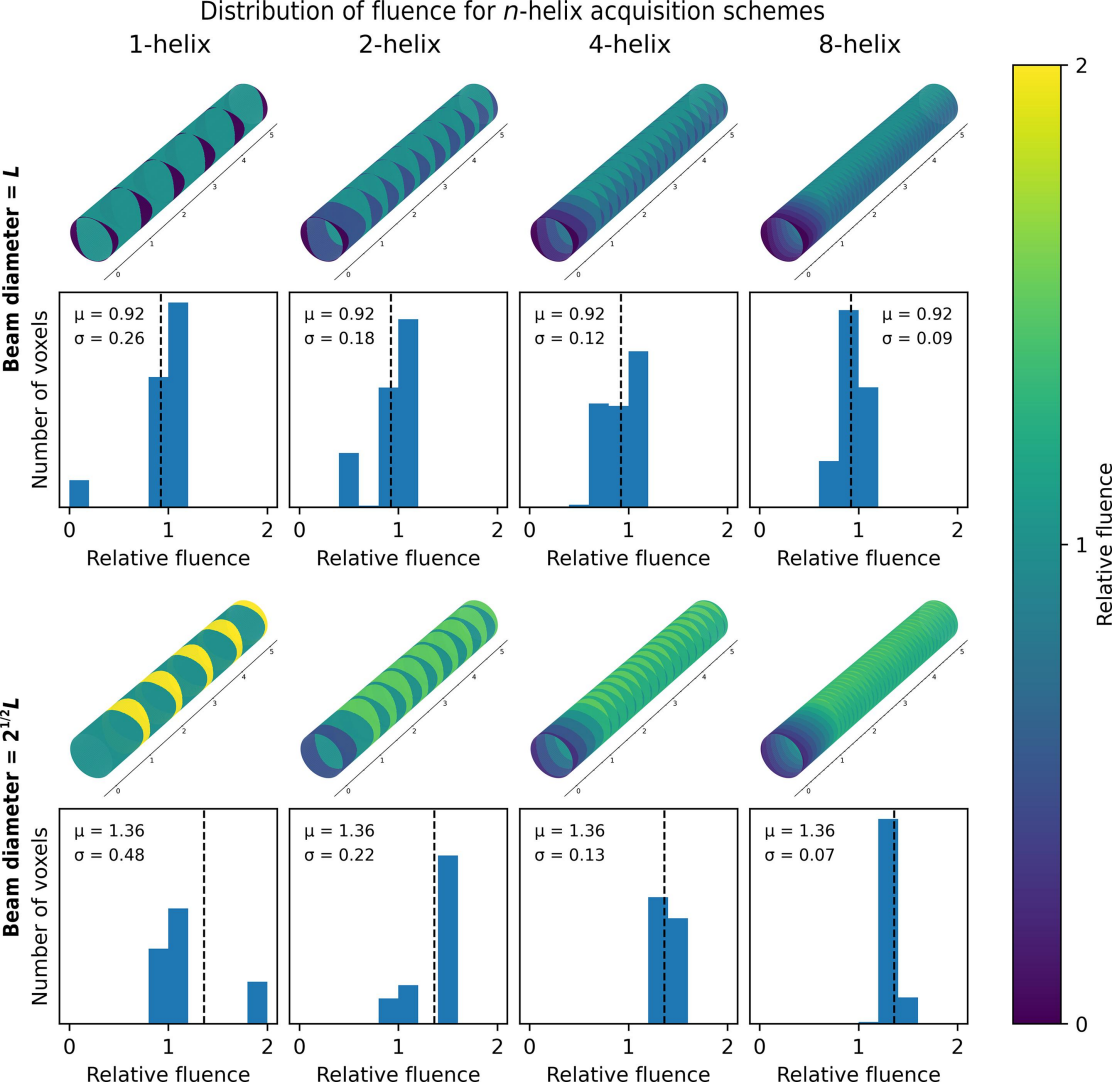
Fluence distribution for the *n*-helix tilt scheme with a beam size of *L* equal to the edge length of the square detector (top) and a beam size of 2^1/2^ *L* that exactly covers the square field of view (bottom). For each beam size, the distribution of fluence across the pillar is shown above the histogram of voxel values.

Fig. 9 ▸ shows the distribution of fluence for the *n*-helix data-acquisition strategies as a function of *n* for two possible beam sizes. The optimal beam size is one which has a diameter equal to the shortest edge length of the detector such that the beam is fully enclosed by the data-collection area. For a square detector of side length *L* this will be a round beam with a diameter of *L*. More typically, a beam size with radius equal to the distance from the centre of the data-collection area to the corners of the detector may be used. For a square detector of side length *L* this will be a round beam with a diameter of 2^1/2^ *L*. It should be noted that if a circular beam is used with an area larger than the field of view of the detector, there will always be areas of the sample which receive additional fluence from adjacent data-acquisition positions without an additional transfer of information. This means that for a circular beam with a diameter of >*L* some information about the sample will always be lost.

For a beam size of *L*, it can be seen that for the one-helix strategy parts of the sample will receive a relative fluence of 1, corresponding to the total fluence of a single tilt series; however, since for this beam size the corners of the detector will not be illuminated, some areas of the sample may receive zero fluence. This means that no information will be transferred about these parts of the sample. However, it should be noted that at commonly used pixel sizes and pillar thicknesses the corners of the detector should typically contain very little sample if the pillar is properly aligned and oriented parallel along a detector edge. Therefore, it is still possible to ensure that only a minimal amount of information is lost.

For a beam size of 2^1/2^ *L*, it can be seen that for the one-helix strategy parts of the sample will receive a relative fluence of 1, corresponding to the total fluence of a single tilt series; however, a significant area of the sample will receive twice this fluence. For those parts of the sample receiving a relative fluence of 2, for every electron that results in useful signal there is another electron that only causes damage to the sample, with no transfer of information. For both beam sizes, as the amount of overlap between the sweeps increases, as defined by the *n*-helix parameter, the distribution of fluence is increasingly uniformly distributed across the sample, while the mean fluence received by the sample remains constant. This is also illustrated by the histograms of the voxel fluence distributions. As the *n*-helix parameter increases, the variance of the fluence distribution decreases, with the variance for the eight-helix being much lower than for the one-helix strategy.

For a circular beam of size 2^1/2^ *L*, the mean fluence for higher order *n*-helix strategies tends to a value of 1.36 across the sample. This means that for this beam size, the *n*-helix data-acquisition strategy will always result in a higher exposure than a single tilt series, which is a natural consequence of having overlapping regions. In practice, this would mean that to give a final electron fluence of 136 e^−^ Å^−2^, each tilt-series acquisition should be configured to have a lower fluence of 100 e^−^ Å^−2^. Set against this is that there will always be some damage done to the sample by electrons which do not contribute useful signal during the data collection.

**Figure 10:**
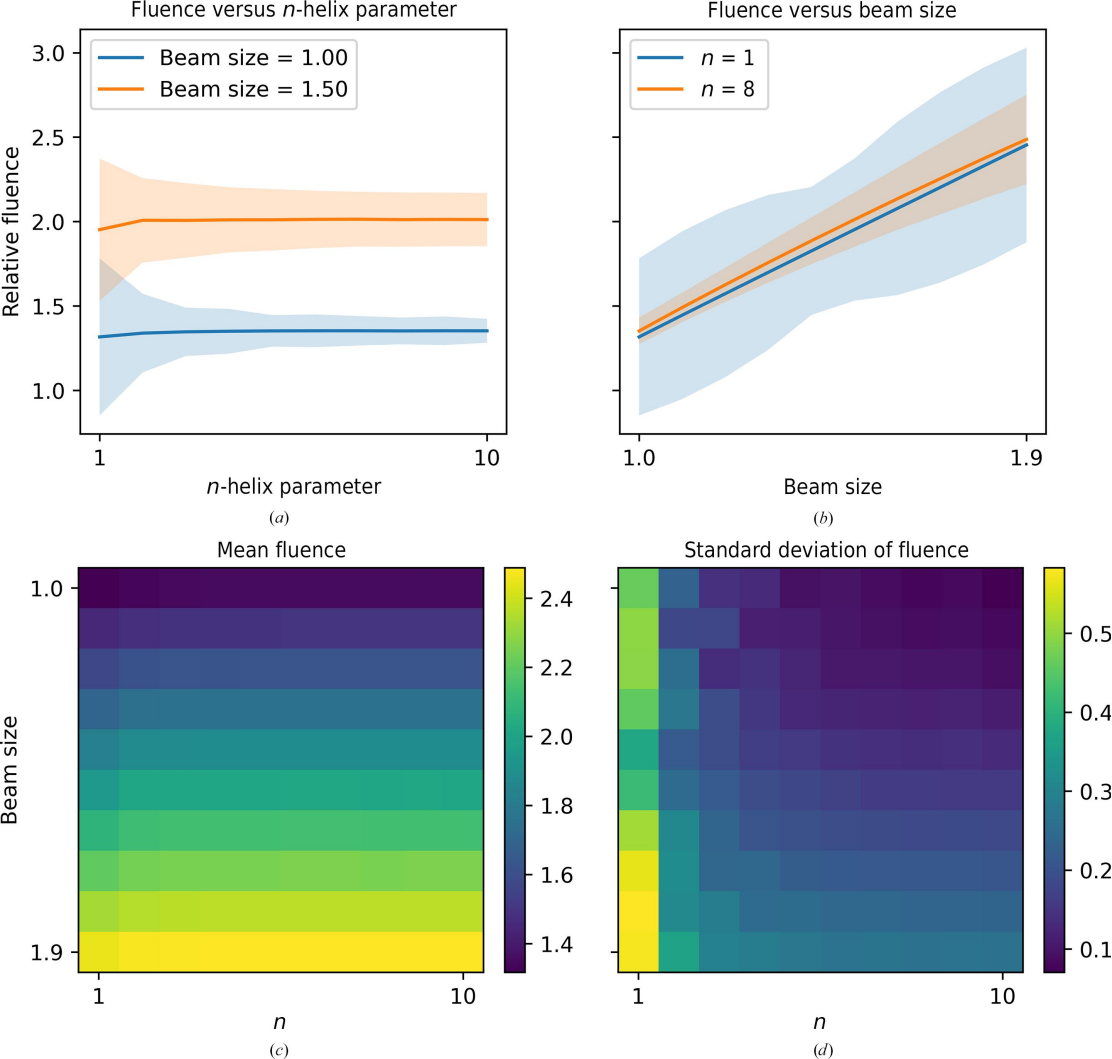
Fluence distribution for the *n*-helix strategy. (*a*) Relative fluence as a function of the *n*-helix parameter for a circular beam of size 2^1/2^ *L* that exactly covers the square field of view (beam size = 1) and a beam size 1.5 times larger. (*b*) Relative fluence as a function of the beam size for a one-helix and an eight-helix strategy. (*c*) Mean relative fluence as a function of both beam size (*y* axis) and *n*-helix parameter (*x* axis). (*d*) Standard deviation of the relative fluence as a function of both beam size (*y* axis) and *n*-helix parameter (*x* axis).

In some cases, it may be necessary to use a beam size larger than the optimal beam size. Fig. 10 ▸ shows the mean fluence and the standard deviation of the fluence across the sample as a function of the *n*-helix parameter for a beam size of 2^1/2^ *L* (which we define as having beam size = 1) and a beam size 1.5 times larger. Fig. 10 ▸(*b*) shows the mean fluence and the standard deviation of the fluence across the sample as a function of the beam size for a one-helix strategy and an eight-helix strategy. The mean and standard deviation of the relative fluence are also shown as a function of both the beam size and the *n*-helix parameter in Figs. 10 ▸(*c*) and 10 ▸(*d*), respectively. The mean fluence remains constant as a function of *n*-helix parameter for a given beam size, while the standard deviation decreases. However, the mean fluence increases linearly with beam size, with the standard deviation also increasing. Whilst the mean fluence for a beam size of 2^1/2^ *L* is ∼1.36 times the fluence for a single tilt series, the mean fluence for a beam size 1.5 times this is ∼2 times the fluence for a single tilt series. This would mean that for a beam size 1.5 times larger than required to cover the data-acquisition area, half of the electrons causing damage on the sample would not be detected. Therefore, reducing the beam size is key to ensuring the transfer of high-resolution information using the *n*-helix data-acquisition strategy.

### 3.7. Reconstruction quality

**Figure 11:**
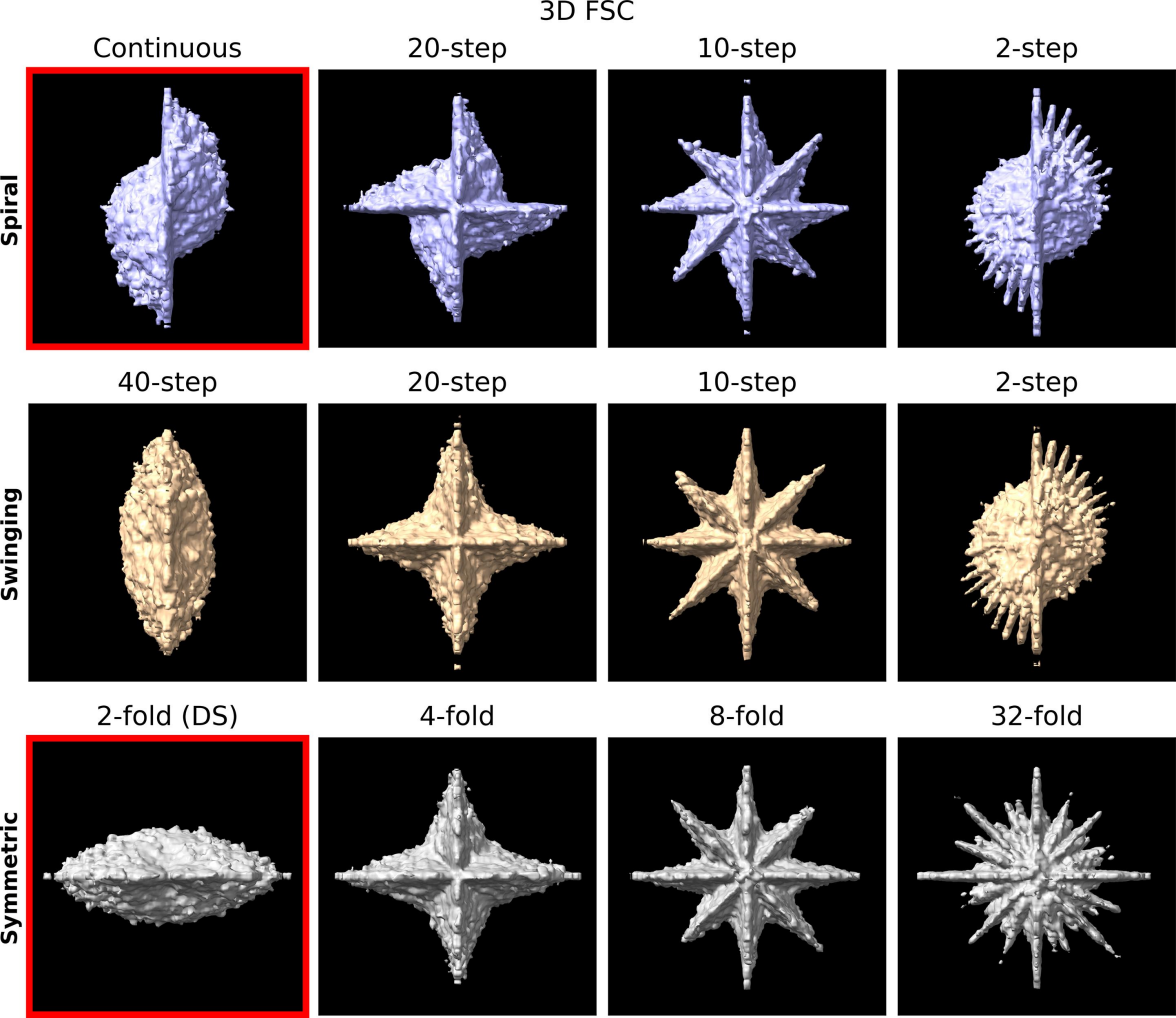
3D FSC at the 70% level for spiral (top), swinging (middle) and symmetric (bottom) schemes. The figure is oriented to show the 3D FSC along the *XZ* plane. Four tilt schemes from each family are shown with symmetry increasing from left to right. The continuous and classic symmetric (DS) schemes are outlined in red. The Nyquist limit is 3 Å.

To assess the reconstruction quality obtained by using the different tilt schemes, a number of synthetic data sets were produced. In these data sets, 8000 ribosome particles were simulated with a preferred orientation, such that the atomic coordinates from the input PDB file were merely translated randomly within the sample volume. The synthetic data sets were then reconstructed and the particles were averaged. The 3D FSC was then calculated by computing the FSC in local regions of Fourier space, as shown in Fig. 11 ▸ for each of the tilt schemes. The 3D FSC shows the same distribution of information transfer as shown previously in Fig. 5 ▸, with the higher symmetry tilt schemes having a more isotropic information transfer.

**Figure 12:**
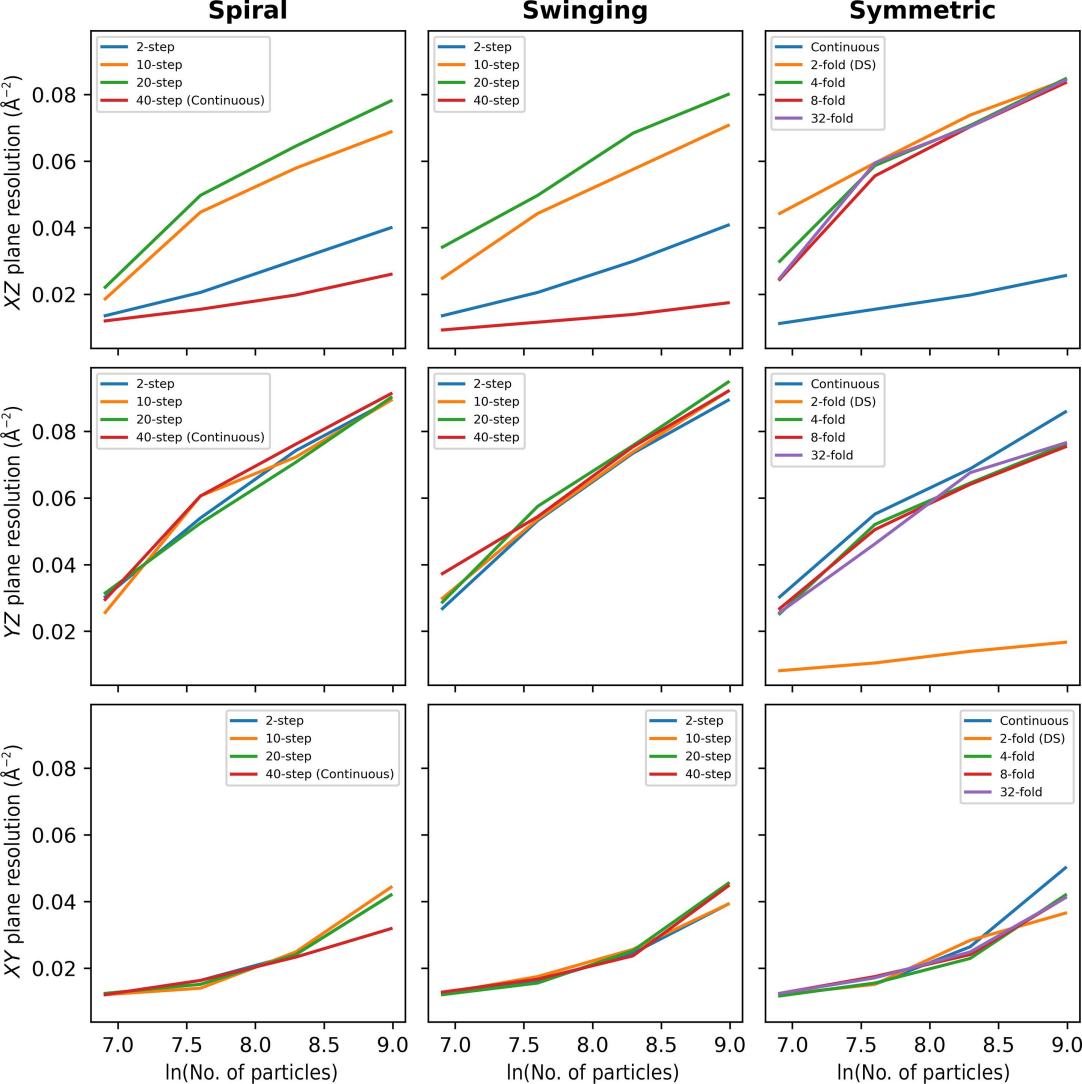
Rosenthal–Henderson plots of the directional resolution in the *XZ* (top), *YZ* (middle) and *XY* (bottom) planes as a function of number of particles for the spiral (left), swinging (centre) and symmetric (right) schemes. Continuous and classic dose-symmetric (DS) schemes are highlighted.

The FSC was also calculated as a function of the number of particles in the *XY*, *XZ* and *YZ* planes. Fig. 12 ▸ shows Rosenthal–Henderson plots of the directional resolution as a function of the logarithm of the number of particles. This shows that for the spiral family of tilt schemes, the directional resolution is best for the ten-step and 20-step schemes, with the other schemes having lower resolution in the *XZ* plane. For the swinging tilt schemes, the ten-step and 20-step schemes also show the best directional resolution, again with better information transfer in the *XZ* plane. The 20-step scheme corresponds to the fourfold-symmetric scheme and the ten-step scheme corresponds to the eightfold-symmetric scheme. Finally, for the symmetric tilt scheme family, the fourfold, eightfold and 32-fold schemes show the most consistent directional resolution. The classic (twofold) symmetric scheme has lower resolution in the *YZ* plane.

## 4. Conclusions

New developments in hardware and sample preparation will enable the fabrication of pillar-shaped specimens for cryo-ET from beam-sensitive biological samples. We have shown that this will provide significant benefits for tomographic data acquisition and reconstruction without the missing-wedge artefacts that are inherent in tomography of planar samples using a limited tilt range. However, there are a number of potential issues inherent in the production of pillar samples and their subsequent data acquisition. It has been shown that an unavoidable damage layer is acquired during FIB milling of planar samples (Berger *et al.*, 2022 ▸; Parkhurst *et al.*, 2023 ▸). Hence, care needs to be taken to ensure minimal damage to biological structures during the milling of pillar samples. During data acquisition, charging may cause beam deflection or even deformation of the pillar sample itself under certain circumstances. This may be mitigated through the judicious application of metal sputter coating (Schaffer *et al.*, 2017 ▸; Beale *et al.*, 2020 ▸); however, the sample-preparation conditions will need to be optimized. It should also be noted that since pillar samples will need to be prepared such that there is a single pillar per cryo-EM grid, pillar samples will likely have lower throughput than planar samples, where a large number of lamellae can be prepared on a single grid.

The classic dose-symmetric scheme has become the standard for planar samples, as higher tilt angles contribute less information due to the increase in the effective thickness of the sample. For pillar samples with perfect cylindrical or conical geometry, views from all angles of the sample will contribute the same amount of information; however, beam damage will still be an issue, with the first images acquired transferring more high-resolution information than the last images. Therefore, the order of data acquisition will still be important and for beam-sensitive samples it may be necessary to spread the fluence to achieve isotropic information transfer. Use of a pillar geometry is not strictly necessary for a typical STA workflow; however, isotropic resolution with no missing wedge is desirable for imaging large-volume cellular samples, vesicles, organelles or bacteria, and for performing analysis of lysis events and pore-forming proteins (Phillips *et al.*, 2021 ▸). Isotropic information transfer will also aid algorithms which attempt to inpaint high-resolution information (Bellos *et al.*, 2019 ▸).

Three families of related tilt schemes have been evaluated to assess the benefit of using alternative data-acquisition schemes other than a continuous tilt scheme or the classic dose-symmetric scheme. These schemes can be combined with an *n*-helix tilt scheme where beam shifts are combined with overlapped interleaved tilt series to reconstruct the entire volume of the pillar, maximizing the utility of the pillar geometry for large-volume cryo-ET. Importantly, once the desired angular sampling has been specified, the total electron budget can be split amongst *n* interleaved tilt series. When using the *n*-helix acquisition scheme, tiling a circular beam profile with the rectangular field of view results in areas of the sample being illuminated, and therefore damaged, without transferring any additional information. A similar issue is seen in montage tomography of planar samples. Rectangular condenser apertures can reduce the area of the sample outside the acquisition area being illuminated, effectively addressing this issue (Chua *et al.*, 2023 ▸). There will be a trade-off in complexity for both circular and rectangular apertures when using the *n*-helix acquisition strategy. On the one hand, the *n*-helix strategy will allow the entire volume to be reconstructed as a single object, reducing the computational burden and associated artefacts of correlation and merging of multiple 3D volumes, and will allow the fluence to be more uniformly distributed across the sample. On the other hand, the reconstruction will be more complex: in STA, where high-resolution information is important, dose-weighting is applied; this will be complicated for the *n*-helix strategy where, as *n* increases, the number of different fluences will also increase.

We have shown that a continuous tilt scheme may result in anisotropic information transfer for beam-sensitive biological samples due to the limited ability to fractionate the fluence across many images. Higher order dose-symmetric schemes can result in a more uniform distribution of fluence at the expense of greater complexity in the data acquisition. In this regard, hardware limitations may hinder the ability to execute arbitrarily complex tilt schemes. However, higher order dose-symmetric schemes are no more demanding on the mechanics of the tilt stage than the classic dose-symmetric scheme which is used regularly to collect high-resolution cryo-ET data. The more uniform information transfer also comes at the cost of a larger jump in fluence between adjacent tilt images, which could result in alignment issues due to fluence-induced sample deformation. The higher the symmetry, the larger the maximum jump in fluence between adjacent images.

The analysis described here focused on an idealized pillar sample which has a constant circular cross section over the length of the pillar. However, in practice, the pillar may have a conical geometry, with a tapered circular cross section which is thinner at the end of the pillar than at the base. In this case, the data-collection strategies described here would be unaltered. In contrast, where the pillar cross section is not circular it may be preferable to match the symmetry of the scheme to the symmetry of the pillar cross section. As an extreme example, for a square cross section the fourfold scheme would be preferred.

The fourfold dose-symmetric scheme is more efficient than the classic dose-symmetric scheme in terms of the total accumulated stage tilt, requiring 75% less total stage rotation, and only has a marginally higher maximum fluence jump between adjacent images. It also produces a much more isotropic transfer of information. A practical approach, therefore, may be to use the continuous tilt scheme when the sample is not beam-sensitive or isotropic information transfer is not necessary and to use the fourfold dose-symmetric scheme if the sample is beam-sensitive and isotropic information transfer is desirable. Future work will focus on experimental validation of these results once the routine production of biological pillar samples is possible.

## 5. Related literature

The following reference is cited in the supporting information for this article: Grandi (1728 ▸).

## Supplementary Material

Supplementary information including Supplementary Figures. DOI: 10.1107/S2059798324004546/ai5012sup1.pdf

## Figures and Tables

**Table 1:** Simulation parameters

Parameter	Description	Value
*E*	Energy	300 keV
Δ*f*	Defocus	2.5 µm
Cs	Spherical aberration	2.7 mm
Cc	Chromatic aberration	2.7 mm
Δ*I*/*I*	Current spread	0.33 p.p.m.
Δ*V*/*V*	Voltage spread	0.80 p.p.m.
Δ*E*	Energy spread	0.8 eV
θ_c_	Source spread	0.05 mrad
*d*_px_	Pixel size	1 Å
*t*_s_	Multislice *z*-slice thickness	5 Å
	Potential approximation	Lobato & Van Dyck (2014 ▸)
*S*_E_	Beam-damage sensitivity coefficient	0.04 Å^4^/e^−^
*D*_E_	Total No. of incident electrons	140 e^−^ Å^−2^
*N*	No. of images per tilt series	40
