# Supplementary material for: Pillar data-acquisition strategies for cryo-electron tomography of beam-sensitive biological samples
Source: Acta Crystallogr D Struct Biol. 2024 Jun 3;80(Pt 6):421–38. doi: 10.1107/S2059798324004546 (PMC11154591; doi:10.1107/S2059798324004546)
Supplement: Supplementary file 1 [file d-80-00421-sup1.pdf]

STRUCTURAL BIOLOGY

Volume 80 (2024)

Supporting information for article:

Pillar data-acquisition strategies for cryo-electron tomography of beam-sensitive biological samples

James M. Parkhurst, Trond Varslot, Maud Dumoux, C. Alistair Siebert, Michele Darrow, Mark Basham, Angus Kirkland, Michael Grange, Gwyndaf Evans and James H. Naismith

S1. Schematics for construction of extended tilt schemes

Figures S1, S2 and S3 show schematics illustrating the construction of the spiral, swinging and symmetric tilt schemes, respectively. In each case, the tilt range is from –90° to +90° but in these figures, the tilt step is large (11.25°) for illustration purposes. The spiral scheme, (Figure S1), starts from the continuous acquisition scheme. For the 2-step spiral scheme, each pair of tilt angles is de-interleaved to produce 2 sweeps which are then concatenated to produce the final ordering of tilt angles. Spiral schemes with larger step sizes are formed in the same way, for example the figure shows how the 4-step spiral scheme is formed by again starting with the continuous scheme and de-interleaving sets of 4 tilt images to produce 4 sweeps which are then concatenated to produce the final ordering of tilt angles.

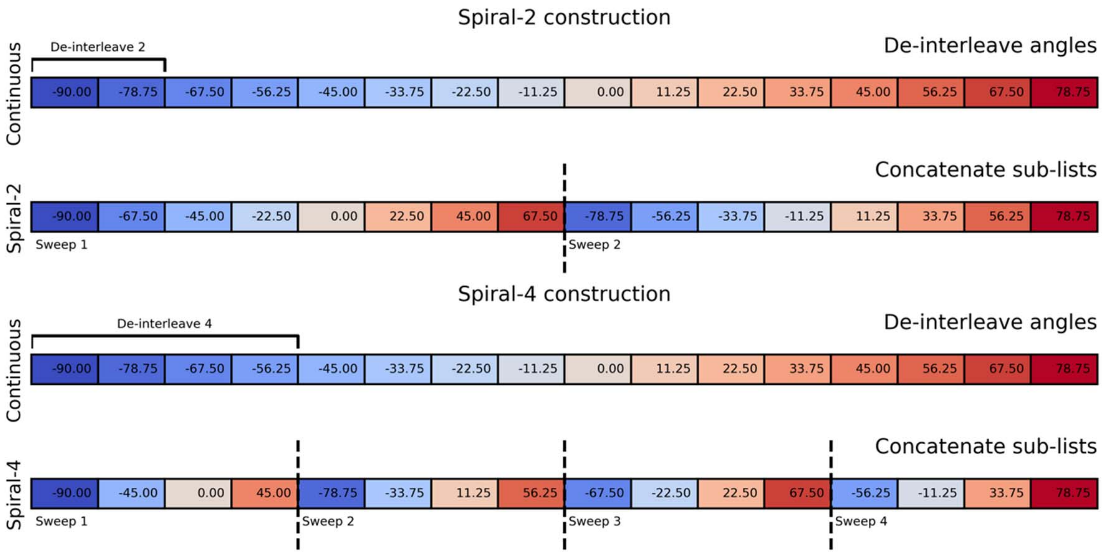

Figure S1 Construction of a spiral tilt scheme. Starting with a list of angles for the continuous tilt scheme, the list is de-interleaved to produce a set of tilt series all moving from negative to positive tilt angle.

The swinging scheme, (Figure S2), also starts from the continuous acquisition scheme. For the 2-step swinging scheme, each pair of tilt angles is de-interleaved to produce 2 sweeps where the second sweep is acquired in reverse. The sweeps are then concatenated to produce the final ordering of tilt angles. Swinging schemes with larger step sizes are formed in the same way, for example the figure shows how the 4-step swinging scheme is formed by again starting with the continuous tilt scheme and de-interleaving sets of 4 tilt images to produce 4 sweeps with the even sweeps acquired in reverse. The sweeps are then concatenated to produce the final ordering of tilt angles.

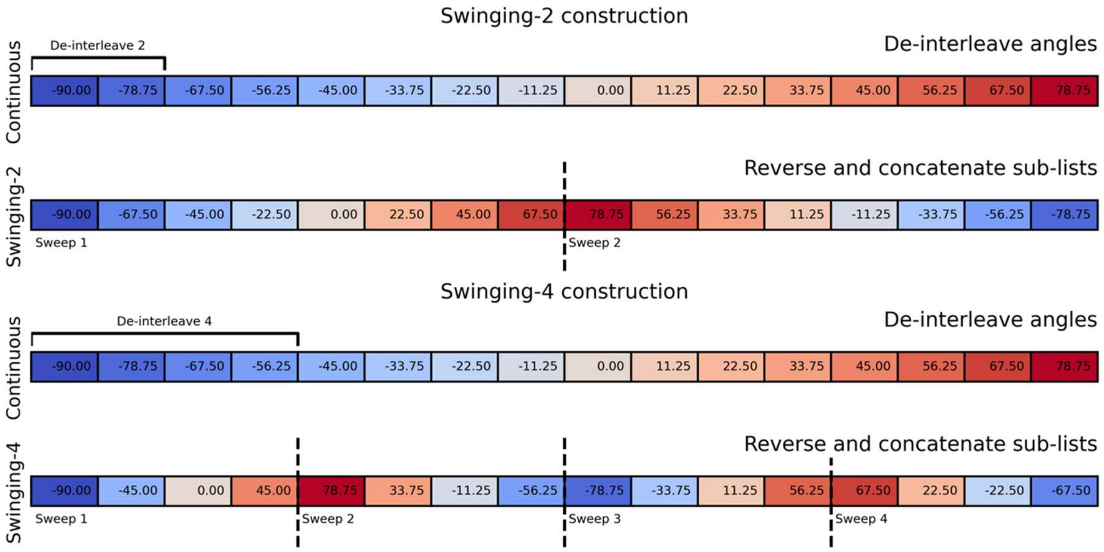

Figure S2 . Construction of the swinging tilt scheme. Starting with a list of angles for the continuous tilt scheme, the list is de-interleaved to produce a set of tilt series that alternate between moving from negative to positive, then positive to negative tilt angle.

Finally, the symmetric scheme, (Figure S3) starts from the classic dose-symmetric (2-fold) acquisition scheme. Higher symmetry schemes are then constructed recursively. For the 4-fold symmetric scheme, the list of angles in the 2-fold scheme is split in two down the middle as shown in the figure resulting in two sub lists. The second sub list is then reversed and the two sub lists are interleaved in sets of 1 tilt angle to produce the new tilt angle order. To produce the 8-fold scheme, the same procedure is applied to the 4-fold symmetric tilt scheme except that the two sub lists are interleaved in sets of 2. This procedure can be extended to higher order symmetric tilt schemes.

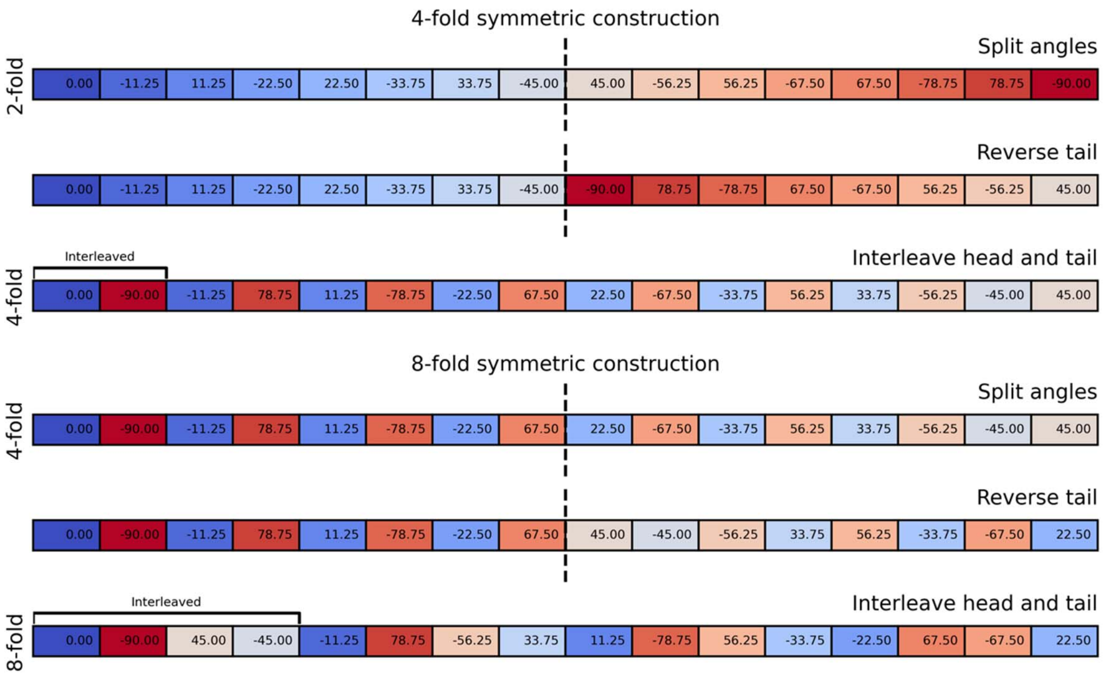

Figure S3 Construction of the symmetric tilt scheme. Starting from the classic dose-symmetric tilt scheme, the list of angles is split in two, the tail is then reversed, and the two subsets of angles are then interleaved. To produce higher-order symmetry schemes, this process is repeated recursively.

S2. Order of image acquisition

Figure S4 illustrates the order of image acquisition for the different tilt schemes. The top row shows the acquisition order for selected spiral tilt schemes, including the continuous tilt scheme which is highlighted in red. The middle row shows the acquisition order for selected swinging tilt schemes. Finally, the bottom row shows the acquisition order for selected symmetric tilt schemes, including the classic (2-fold) dose-symmetric scheme which is also highlighted in red. Tilt angles acquired early in the data acquisition are coloured dark and those collected later in the data acquisition are coloured lighter. The diagram highlights the effective symmetry of the tilt schemes; the diagrams for the large step size swinging schemes and symmetric schemes have both rotational and mirror symmetry, whereas the spiral schemes only have rotational symmetry.

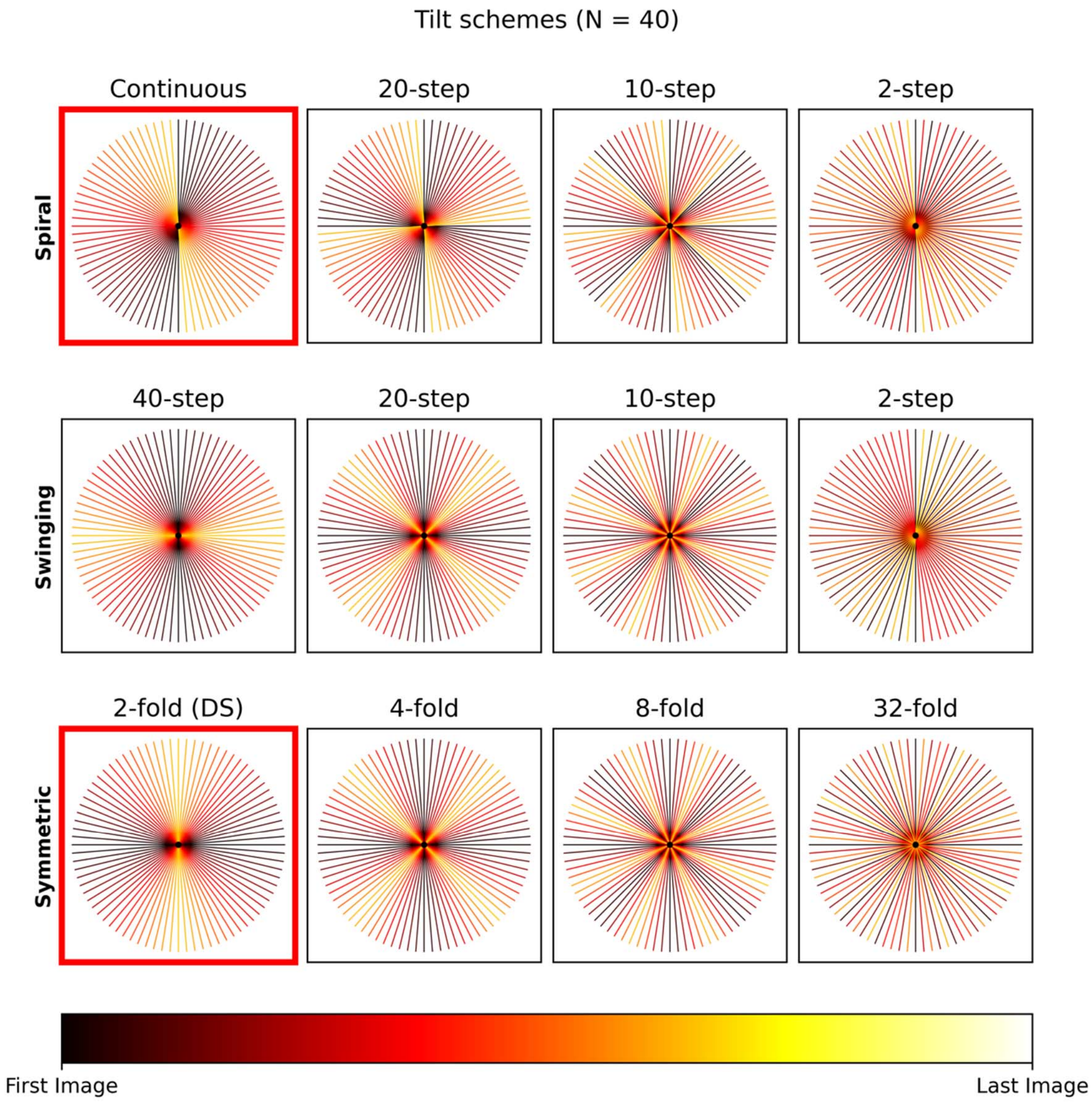

Figure S4 Order of image acquisition for the spiral (top), swinging (middle) and symmetric (bottom) schemes. Four tilt schemes from each family are shown with symmetry increasing from left to right. The continuous and classic dose-symmetric (DS) schemes are highlighted in the plots outlined in red. The order of image acquisition is illustrated by the colour of the tilt angles going from dark to light.

S3. Angular distribution of B factors

Figure S5 shows a polar plot with a radius at an angle given by the B-factor: $r(\theta_n) = nB$. These highlight the angles which will be subject to the most beam damage. For the continuous tilt scheme, at –90° the B-factor is zero and the B-factor increases with tilt angle until it reaches its maximum at +90°. For the spiral family of tilt schemes, the effect of the step size is to change the nature of the spiral distribution of the B-factor. For example, the 2-step scheme forms a spiral with a reduced pitch. Increasing the number of steps

further decreases the pitch of the spiral until the spiral becomes dominated by the angular sampling equivalent to the finite number of tilt images. For the swinging samples, a similar pattern is observed. At large step size, the swinging schemes produce an angular distribution of B-factors that has both mirror and rotational symmetry. For the symmetric family, increasing the symmetry results in a B-factor distribution similar to a Rose curve (Grandi, 1728) with an even number of petals.

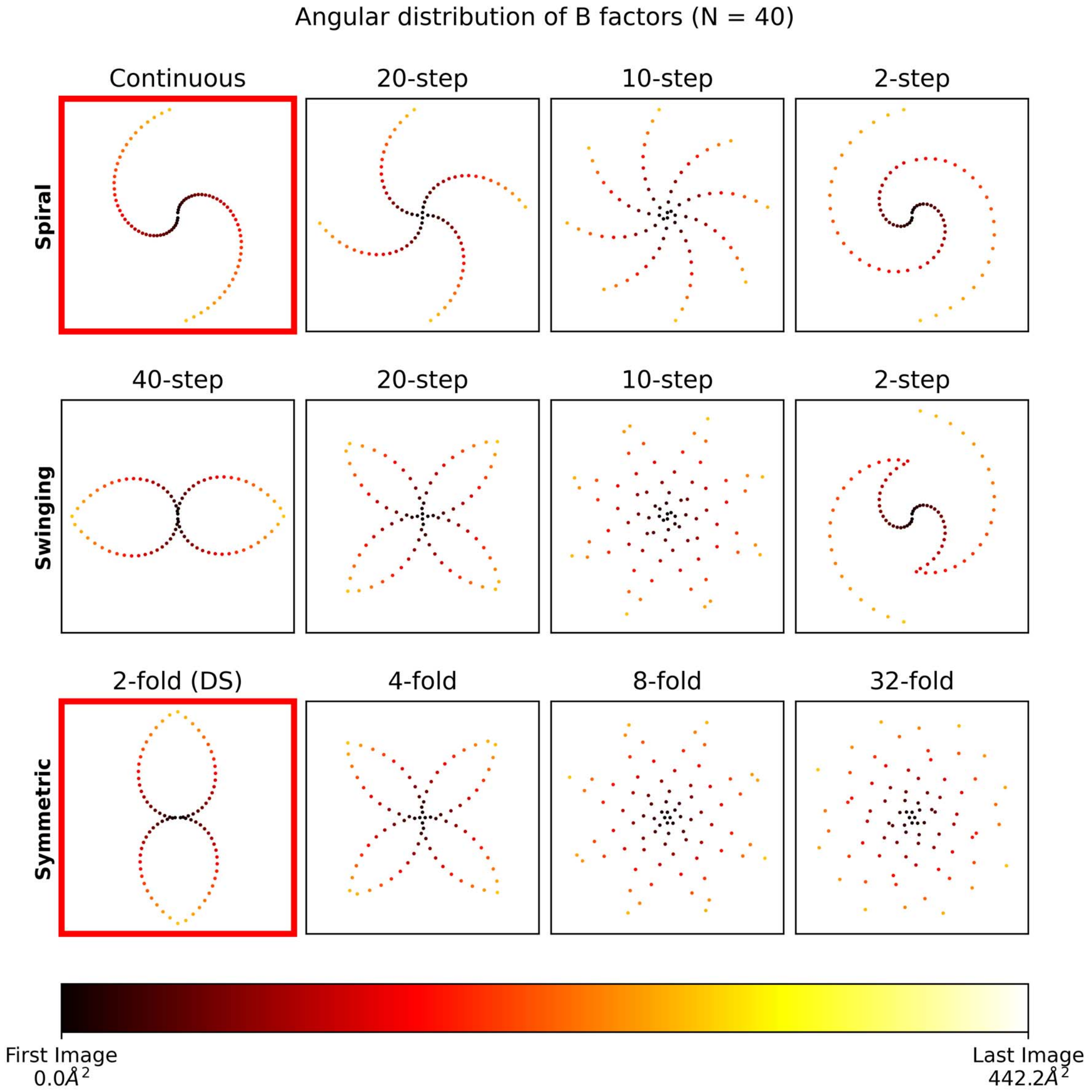

Figure S5 Polar plots showing the angular distribution in Fourier space of the relative B-factor for spiral (top), swinging (middle) and symmetric (bottom) schemes. The x-axis corresponds to the direction orthogonal to the beam and the y-axis corresponds to the direction parallel to the beam. Four tilt schemes from each family are shown with symmetry increasing from left to right. The continuous and classic dose-symmetric (DS) schemes are highlighted in red. The order of image acquisition is illustrated by the colour

of the tilt angles going from dark to light. The relative magnitude of the B-factors is illustrated by the radius of the point at each tilt angle.

S4. Fluence distribution for the *n*-helix tilt scheme with different beam diameters

For a circular beam and a square data collection area, if the beam fully covers the data collection area, the beam will irradiate the sample outside the data collection area. The optimal beam size is the circular beam profile which has a diameter equal to the shortest edge length of the data collection area. Figure 6 in the main article shows the electron fluence distribution for a beam size equal to the edge length of the detector and the smallest beam size which fully covers the data collection area. Figures S6 and S7 show the electron fluence distribution in pillar samples with beam sizes 1.5 times and 1.9 times the beam size required to fully cover the detector, respectively. The 3D fluence distribution plots show that, as the beam sizes increases, so does the maximum fluence received by parts of the sample; furthermore, the histograms show that both the mean and the variance in the relative fluence across the sample also increase as the beam size increases.

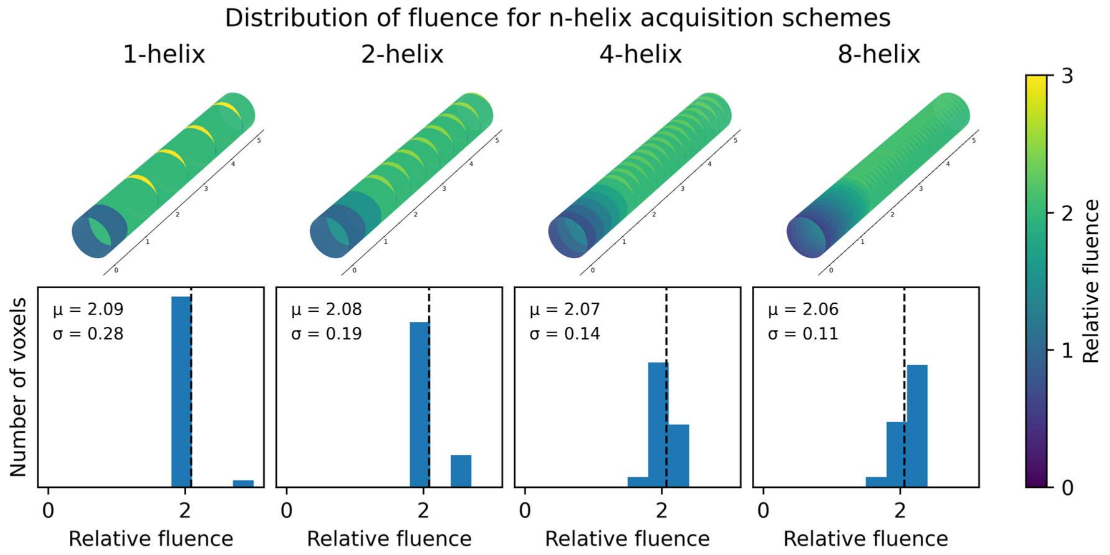

Figure S6 Fluence distribution for the *n*-helix tilt scheme with beam size = 1.5 times the beam size required to fully cover the detector. The top row shows the distribution of fluence across the pillar. The bottom row shows histograms of voxel values.

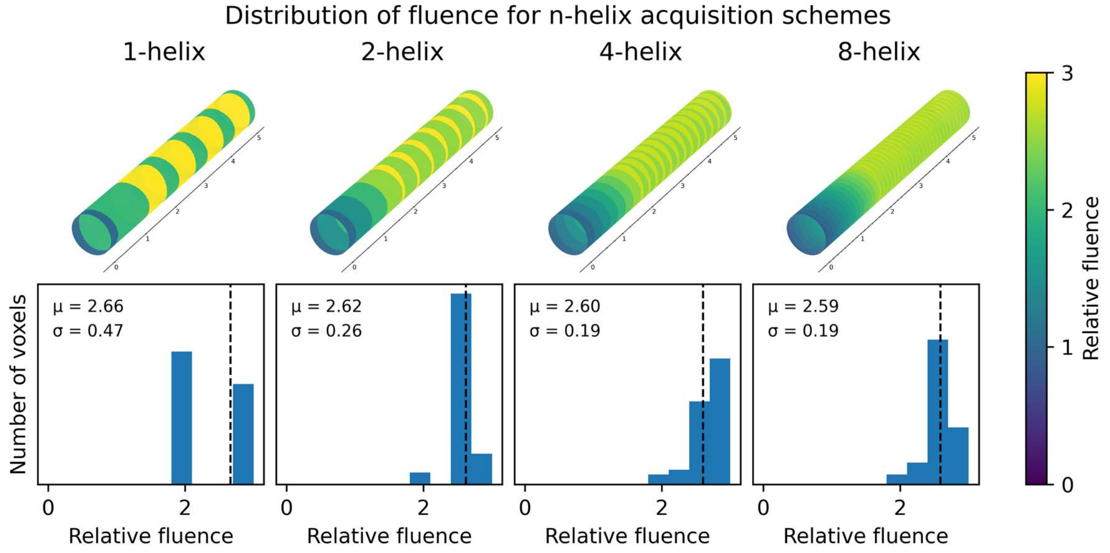

Figure S7 Fluence distribution for the *n*-helix tilt scheme with beam size = 1.9 times the beam size required to fully cover the detector. The top row shows the distribution of fluence across the pillar. The bottom row shows histograms of voxel values.

S5. Simulated images

Figure S8 shows an example of simulated micrograph of a pillar sample. The scale bar indicates the size of the pillar which was simulated to have a diameter of 300 nm. The pillar contains 1000 ribosome particles within the 0.04 μm^3 volume, giving a density of 23579 ribosomes / μm^3.

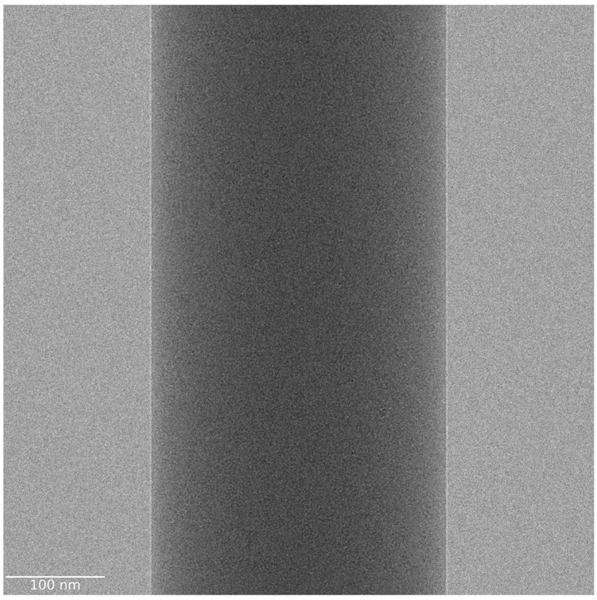

Figure S8 Simulated micrograph of a pillar sample containing Ribosomes.

References

Grandi, G. (1728). Flores geometrici ex Rhodonearum, et Cloeliarum curvarum descriptione resultantes. Tartinium & Franchium. https://archive.org/details/bub_gb_UBqguM3wdwQC.
